# Discovery of novel murine PML isoforms

**DOI:** 10.1080/19491034.2026.2646815

**Published:** 2026-03-23

**Authors:** Karolína Anderová, Lenka Horníková, Vojtěch Šroller, Boris Ryabchenko, Dalibor Pánek, Prokopis C. Andrikopoulos, Jiří Zahradník, Jitka Forstová, Sandra Huérfano

**Affiliations:** a Department of Genetics and Microbiology, Faculty of Science, Charles University, BIOCEV, Vestec, Czech Republic; b Imaging Methods, Core Facility, Faculty of Science, Charles University, BIOCEV, Vestec, Czech Republic; c First Faculty of Medicine, Charles University, BIOCEV, Vestec, Czech Republic

**Keywords:** PML, PML isoforms, PML nuclear bodies, Arsenic trioxide, Mouse PML isoforms

## Abstract

Promyelocytic leukemia protein (PML) orchestrates the formation of PML nuclear bodies (PML NBs), membraneless organelles with diverse regulatory roles. Despite their importance, the specific functions of individual PML splicing variants remain unclear, particularly in murine models. Here we study the repertoire of murine PML isoforms expressed in mouse tissues and cells. We demonstrate that in addition to canonical mPML1–3, mice express five predicted variants (mPMLX1, mPMLX2, mPMLX4–X6) and a novel isoform, mPMLX7, distinguished by unique RBCC domain splicing. All isoforms exhibit distinct turnover kinetics at endogenous PML NBs. In PML-knockout cells, all isoforms except mPMLX7 form NBs de novo and are degraded upon arsenic exposure. Molecular dynamics simulations suggest mPMLX7 adopts a stable conformation; furthermore, this isoform is enriched in the nucleoplasm, suggesting a specialized function. Altogether, this isoform-resolved PML system provides a relevant model for dissecting the wide spectrum of PML-associated processes.

## Introduction

The promyelocytic leukemia protein (PML) was originally identified in patients with a type of acute promyelocytic leukemia (APL) as part of an oncogenic fusion with retinoic acid receptor alpha (RARα), resulting from chromosomal translocation t(15;17) [1–4]. In normal cells, PML serves as a scaffold protein within multiprotein structures known as PML nuclear bodies (PML NBs) [5]. In APL, the chimeric PML-RARα oncoprotein is aberrantly dispersed throughout the nucleus and cytoplasm, thereby impairing PML function and blocking promyelocytic differentiation [4,6].

Since its discovery as a tumor suppressor in the 1990s, PML has been extensively studied and linked to diverse cellular processes, including angiogenesis, cell cycle regulation, apoptosis, senescence, DNA damage responses, protein degradation, and antiviral responses [7–12]. Interestingly, PML NBs can exert opposing functions depending on their intra- and extracellular environment [13].

PML NBs are spherical membraneless organelles found in most mammalian cells, and are nonrandomly associated with the nuclear matrix [14–16]. Depending on the tissue and cell type, the stage of differentiation, the cell cycle phase, and stress conditions, PML NBs can range in diameter between 0.2 and 1 μm and are found in quantities ranging from 1 to 30 bodies per nucleus [7,12]. PML forms an insoluble outer ring-like shell enclosing a core of several stable or transient partner proteins. To date, 271 proteins have been identified as PML NB interaction partners, with SP100, Daxx, and SUMO being the best characterized [16–19]. PML NBs are typically free of nucleic acids; however, they have been shown to dynamically interact with transcriptionally active sites of chromatin, playing a role in the regulation of genome function [20–23].

The broad spectrum of currently identified interaction partner hints at the potential molecular mechanisms underlying the diverse functions of PML NBs. It has been proven that these bodies act as centers for transient protein deposition within specific nuclear regions, where post-translational modification (PTMs) and specific protein interactions facilitate distinct cellular processes [7,16,24–26]. Furthermore, given that PML protein exists in various isoforms, increasing evidence suggests that individual PML isoforms are crucial factors influencing the composition and functions of PML NBs [13,16,17].

In humans, the alternative splicing of nine *PML* exons produces mRNAs encoding seven major isoforms (hPMLI to hPMLVII), six nuclear and one cytoplasmic. Although all PML isoforms share an identical N-terminal region, they significantly differ in the composition of their C-terminus. Specifically, exons 1–3, conserved across all variants, encode the RBCC/tripartite (TRIM) domain, composed of a zinc-coordinating RING finger domain, two B-box domains, and a coiled-coil (CC) domain, which is essential for PML homo-multimerization and NB assembly [27–31]. Apart from the conserved RBCC domain, individual isoforms contain various other functional motifs, including a nuclear localization signal (NLS, hPMLI to VI), a SUMO-interacting motif (SIM, hPMLI to V), an exonuclease III-like domain (Exo-III, hPMLI and partly hPMLIV), and a nuclear export signal (NES, hPMLI). In addition, hPMLV contains a putative C-terminal structural α-helix [32–35].

The murine *Pml* gene (*mPml*) was first isolated in 1995 as the most accessible homolog for human *PML* (*hPML*). Both the *mPml* and *hPML* genes share a similar organization, both consisting of nine exons with conserved intron positions. A comparative analysis showed that mPML and hPML proteins share 80% amino acid sequence similarity and 69% identity with more than 90% similarity in functional domains, including the RBCC domain and the NLS [36]. Three mPML isoforms (mPML1 to 3) have been characterized to date, while six additional isoforms have been computationally predicted (mPMLX1 to X6) [36–38]. Like the hPML isoforms, both the confirmed and predicted mPML isoforms share an identical N-terminal structure, but markedly differ in the composition of their C-terminus [27,36–39].

In this study, we investigate the endogenous expression and characteristics of all previously confirmed murine PML isoforms (mPML1 to mPML3) and those predicted by computational modeling (mPMLX1 to X6), focusing on NB formation, assembly dynamics and responses to cellular stress stimuli. In addition, we perform in silico three-dimensional (3D) structure predictions with molecular dynamics simulations. This work aims to establish a new platform that would enable the study of the individual roles of mouse PML isoforms in PML NB-associated processes.

## Materials and methods

### Cell lines

*Pml*^*+/+*^ or *Pml*^*−/−*^ MEFs (generously provided by Dr. Piero Paolo Pandolfi [40,41]), mouse 3T3 fibroblasts (ATCC, CCL-92), mouse 3T6 fibroblasts (ATCC, CCL-96), and mouse mammary gland cells (NMuMG) (ATCC, CRL-1636) were cultured in Dulbecco’s modified Eagle’s medium (DMEM, Merck, Cat. No. D6429) supplemented with 10% fetal bovine serum (Merck, Cat. No. F7524) and an antibiotic-antimycotic solution (Merck, Cat. No. ECM0010) at 37°C in a humidified atmosphere containing 5% CO_2_.

### Mouse organs

This pilot study aimed to profile PML transcript expression across multiple organs using a single 12-week-old female C57BL/6NCrl mouse. No control group or statistical analysis was included, as the goal was to establish baseline organ-specific data.

The mouse was housed under standard conditions (22 ± 2°C, 55 ± 10% humidity, 12-hour light/dark cycle) with ad libitum access to chow and water, and enrichment provided.

The animal was maintained in the facility until tissue collection. No randomization was applied, as only one animal was used. Following euthanasia by cervical dislocation, the liver, spleen, kidney, lung, heart, brain, thymus, and muscle were dissected. Total RNA was extracted from each organ using the NucleoSpin RNA Kit (Macherey-Nagel, Cat. No. 740,955.50), following the manufacturer’s instructions.

Raw data and protocols are available upon reasonable request to the corresponding author.

### Detection of endogenous mPML transcripts by PCR-based assays

To confirm the endogenous transcription of the previously confirmed and predicted PML isoforms and the novel isoform mPMLX7, total RNA was extracted from different cell lines using the NucleoSpin RNA Kit (Macherey-Nagel, Cat. No. 740,955.50) according to the manufacturer’s protocol. cDNA was synthesized with 660 ng of RNA using LunaScript RT Super Mix (New England Biolabs, Cat. No. E3010) as specified by the manufacturer. A no-RT control was included for each isoform. PCR was performed with Phusion High-Fidelity PCR Master Mix with HF Buffer (New England Biolabs, Cat. No. M0531) using 2 μl of cDNA template per 50-μl reaction volume. The cycling conditions were an initial denaturation at 98°C for 30 s, followed by 35 cycles of denaturation at 98°C for 10 s, annealing at 58–64°C for 30 s, and extension at 72°C for 41 s with a final extension at 72°C for 10 min. Isoform-specific primer pairs were designed based on the following NCBI reference sequences: mPML1, NM_008884.5; mPML2, NM_178087.4; mPML3, NM_001311088.1; mPMLX1, XM_006510860.3; mPMLX2, XM_006510861.3; mPMLX3, XM_006510862.5; mPMLX4, XM_006510864.5; mPMLX5, XM_030244128.2; mPMLX6, XM_017313212.3; and the novel mPMLX7 sequence. mHRPT primers were as previously described [42]. Detailed conditions and the full specifications of all the primers used are listed in Supplementary Table S1. Amplicons were separated on 1.5% agarose gels, stained with Midori Green Advance stain (Nippon Genetics, Cat. No. MG04), and visualized using the InGenius 3 system (Syngene).

For the detection of the full-length transcripts of the predicted mPMLX1 to X6 isoforms, as well as the novel isoform mPMLX7, total RNA was extracted from MEFs using the NucleoSpin RNA Kit (Macherey-Nagel, Cat. No. 740,955.50) following the manufacturer’s protocol. cDNA synthesis was performed using either PrimeScript RT Reagent (Perfect Real Time) (TaKaRa, Cat. No. RR037A) or Maxima H Minus Reverse Transcriptase (Thermo Fisher Scientific, Cat. No. EP0753) with gene-specific primers (Supplementary Table S2) and 800 ng of RNA template, according to the manufacturer’s instructions. A no-RT control was included for each reaction. PCR was performed with Phusion High-Fidelity PCR Master Mix with HF Buffer (New England Biolabs, Cat. No. M0531) using 1 μl of cDNA template per 25-μl reaction volume under the following conditions: an initial denaturation at 98°C for 30 s, 35 cycles of denaturation at 98°C for 10 s, annealing at 57–67°C for 30 s, and extension at 72°C for 1 min; and a final extension at 72°C for 10 min. The sequences of the primers used, together with their detailed conditions, are provided in Supplementary Table S2. PCR products were separated on 0.8% agarose gels, stained with Midori Green Advance stain (Nippon Genetics, Cat. No. MG04), and visualized using the InGenius 3 gel imaging and analysis system (Syngene). Specific DNA amplicons were isolated using the GeneJET Gel Extraction Kit (Thermo Fisher Scientific, Cat. No. K0691) and either cloned into the pENTR/D-TOPO vector using the pENTR Directional TOPO Cloning Kit (Invitrogen, Cat. No. K240020) (mPMLX4, mPMLX6, and mPMLX7) and sequenced, or sequenced directly using the BrilliantDye Terminator v3.1 Cycle Sequencing Kit (NimaGen, Cat. No. BRD3) (mPMLX1 to X3, mPMLX5) with BDX64 Dye Enhancing Buffer (MCLAB, Cat. No. BDX) in a 3130xl Genetic Analyzer system (Life Technologies). Standard sequencing reactions were performed for 35 cycles under the following conditions: 96°C for 10 s, 50°C for 5 s, and 60°C for 2.5 min. The reaction profile was modified based on the primer melting temperatures, as listed in Supplementary Table S2.

### Detection of endogenous mPML transcripts by Oxford Nanopore Technologies (ONT) sequencing

To validate the PCR-based detection of endogenous mPML transcripts using ONT sequencing, total RNA was isolated from *Pml*^*+/+*^ MEFs with the NucleoSpin RNA Kit (Macherey-Nagel, Cat. No. 740,955.50) according to the manufacturer’s instructions and RNA quality and concentration was subsequently assessed. Sequencing library was prepared using the cDNA-PCR Sequencing V14 – Barcoding Kit (ONT, Cat. No. SQK-PCB114.24) following the manufacturer’s protocol. Sequencing was performed with the same kit on a PromethION Flow Cell with R10.4.1 chemistry (FLO-PRO114M) using the PromethION 2 device (ONT). Data analysis included processing reads with the Pychopper programme (RRID:SCR_018966, ONT, available from https://github.com/nanoporetech/pychopper.) and quality assessment with FASTQC, followed by alignment to reference transcript sequences using Minimap2 [43] and visualization in the Integrative Genomics Viewer (IGV) [44]. Detailed information on individual steps is provided in the Supplementary Methodology. Raw transcriptome data are published under NCBI BioProject #PRJNA1381475, BioSample #SAMN54106409 as raw sequences in the Sequence Read Archive #SUB15828003.

### Plasmids and oligonucleotides

Expression vectors encoding the mPML isoforms fused with a turbo-RFP tag (mPML-tRFP), or harboring a stop codon introduced at the end of the mPML coding sequence (mPML-S), yielding non-tagged mPML proteins, were used in this study. For mPML-tRFP expression, plasmids were either purchased (OriGene; Eurofins Genomics Blue Heron) or constructed based on their exon composition using entry sequences from commercial plasmids, MEF RT-PCR product sequences (pENTR Directional TOPO Cloning Kit, Invitrogen, Cat. No. K240020), or synthesized gene fragments (GeneScript). To construct mPML-S plasmids, site-directed mutagenesis was employed to introduce the stop codon at the end of the mPML sequence. Detailed information on plasmid construction, including oligonucleotide primers (Integrated DNA Technologies) and gene fragments, can be found in Supplementary Table S3 and S4.

### Antibodies and chemicals

The following primary antibodies were used in this study: mouse monoclonal antibody against mouse PML protein (Merck, Cat. No. MAB3738), rabbit monoclonal antibody against mouse SUMO-1 protein (ABclonal, Cat. No. A19121) and rabbit polyclonal antibody against mouse GAPDH protein (Merck, Cat. No. G9545). The secondary antibodies were donkey anti-mouse conjugated with Alexa Fluor-488 (Thermo Fisher Scientific, Cat. No. A-21202), donkey anti-rabbit conjugated with Alexa Fluor-647 (Thermo Fisher Scientific, Cat. No. A-31573), goat anti-rabbit conjugated with horseradish peroxidase (Bio-Rad, Cat. No. 1,706,515), and goat anti-mouse conjugated with horseradish peroxidase (Bio-Rad, Cat. No. 1,706,516).

A 26 mM stock solution of ATO was prepared in 1 M NaOH, diluted in DMEM and used at a 5-μM concentration for 1–24 h, as indicated in the figure legends.

### Immunofluorescence

For confocal microscopy, *Pml*^*+/+*^ and *Pml*^*−/−*^ MEFs were transfected with individual mPML isoforms using either TurboFect Transfection Reagent (Thermo Fisher Scientific, Cat. No. R0531) or Metafectene Transfection Reagent (Biontex, Cat. No. T020). For TurboFect reverse transfection, 2 μl of the transfection reagent was mixed with 700 ng of plasmid DNA diluted in 100 μl of serum-free DMEM and incubated for 15 min at room temperature (RT). The mixture was distributed in the well of a 24-well plate and covered with 1 × 10^5^ cells in suspension. The cells were incubated at 37°C in a humidified atmosphere with 5% CO_2_ for 24 h. Alternatively, for Metafectene transfection, 2 × 10^5^ cells were seeded in a 12-well plate and grown to 90%–100% confluence. A total of 1 μg of plasmid DNA and 3 μl of Metafectene Transfection Reagent were each diluted in 30 μl of PBS, combined, and incubated for 20 min at RT according to the manufacturer’s instructions. DNA-lipid complexes were added drop-wise to the cultured cells, followed by incubation for 24 h at 37°C in a humidified atmosphere with 5% CO_2_. After incubation, the cells were rinsed with PBS, fixed in 4% paraformaldehyde in PBS for 15 min at RT, and permeabilized with 0.5% Triton X-100 (Roth) in PBS for 5 min at RT. Following three washes with PBS and the blocking of nonspecific binding sites via incubation with 1% BSA in PBS for 30 min at RT, the cells were immunostained with primary antibodies for 1 h or overnight at 4°C or RT, respectively. After three 10-min washes with PBS, the cells were incubated with secondary antibodies for 30 min at RT in the dark. Cell nuclei were stained with 1 μg/ml DAPI in PBS for 90 s at RT. Coverslips were washed with deionized water, air-dried, and mounted in 3 μl of Mount Liquid Antifade mounting medium (Abberior, Cat. No. ABR-MM-2009-2X15ML). Images were acquired using an LSM 880NLO confocal microscope (Zeiss) with a 63× oil immersion objective (NA 1.4) or an I×71 inverted system microscope (Olympus) equipped with a 20× (NA 0.45) or 40× (NA 0.60) air immersion objective.

### The effect of arsenic treatment on mPML NB dynamics

MEFs or 3T3 cells were seeded on coverslips or 6-well plates. *Pml*^*−/−*^ MEFs were transfected with individual mPML isoforms using Cell Line Nucleofector Kit V (Lonza, Cat. No. VCA-1003). Briefly, cell pellets containing 4 × 10^6^ cells were resuspended in 100 μl of Amaxa nucleofector solution, mixed with 5 μg of plasmid DNA, and transfected using Amaxa Nucleofector II (Lonza) with T020 pre-programmed settings. After the pulse, the cells were mixed with 500 μl of RPMI-1640 medium (Merck, Cat. No. R8758), incubated for 15 min at 37°C in humidified atmosphere with 5% CO_2_, and seeded on cover slips or 6-well plates. On the second day, the cells were treated with 5 μM ATO for 1, 6, or 24 h. After treatment, the cells grown on coverslips were washed with PBS and fixed in 4% paraformaldehyde in PBS for 15 min at RT. PML and SUMO proteins were stained using specific antibodies as described above. Cells grown in 6-well plates were washed with PBS and lysed in lysis buffer (Cell Signaling Technology, Cat. No. 9803S) supplemented with a protease and phosphatase inhibitor cocktail (Cell Signaling Technology, Cat. No. 5872S) and 20 mM N-ethylmaleimide [45] (Merck) for 20 min on ice. Cellular debris was removed by centrifugation at 20,000 × g for 20 min at 4°C. Supernatants (15–25 μg) were separated by SDS-PAGE as described below, and PML and GAPDH proteins were stained using specific antibodies. Protein band intensities were determined using ImageJ software [46] and, unless otherwise stated, normalized to GAPDH levels. Fold changes in protein levels were normalized to control cells. Although most western blots analyzed were not cropped, some blots were cropped for presentation purposes.

### Western blotting

For experiments validating the expression of full-length mPML-tRFP/mPML-S protein isoforms, cells seeded in 12-well plates were transfected using Metafectene transfection reagent as described above. Cells were lysed in 100 μl of lysis buffer (Cell Signaling Technology, Cat. No. 9803S) supplemented with a protease and phosphatase inhibitor cocktail (Cell Signaling Technology, Cat. No. 5872S) for 20 min on ice. Cellular debris was removed by centrifugation (20000 × g, 20 min, 4 °C). Supernatants were separated by SDS-PAGE on NuPAGE Bis-Tris Mini Protein Gels (Thermo Fisher Scientific, Cat. No. NP0322BOX) according to the manufacturer’s instructions. Separated proteins were electro-transferred onto a nitrocellulose membrane in NuPAGE Transfer Buffer (Thermo Fisher Scientific, Cat. No. NP0006) at 2.5 mA cm^−2^ for 90 min. The membranes were blocked in 5% nonfat milk in PBS for 45 min. Immunostaining with primary and secondary antibodies was conducted for 1 h and 30 min, respectively. Incubation with each antibody was followed by extensive washing of the membrane in PBS. The protein bands were developed using enhanced chemiluminescence reagent (Thermo Fisher Scientific, Cat. No. 34,095), and the signal was visualized using an Amersham Imager 600 RGB (GE Healthcare). When required, membranes were re-probed as previously described [47].

### Fluorescence recovery after photobleaching

*Pml*^*+/+*^ MEFs were transfected with individual mPML isoforms using the Cell Line Nucleofector Kit V (Lonza) 2–3 days before FRAP experiments. Cell pellets containing 2 × 10^6^ cells were resuspended in 100 μl of Amaxa nucleofector solution, mixed with 2.5 μg of plasmid DNA, and transfected using Amaxa Nucleofector II (Lonza, Cat. No. VCA-1003) with T020 pre-programmed settings. After the pulse, the cells were mixed with 500 μl of RPMI-1640 medium (Merck, Cat. No. R8758), incubated for 15 min at 37°C in a humidified atmosphere with 5% CO_2_, and seeded in 35-mm glass-bottom dishes (MatTek). FRAP experiments were performed using a Nikon-Yokogawa CSU-W1 spinning disk confocal microscope equipped with a Nikon OMS LAPP-VIS galvo-scanning photo stimulation device. A Nikon CFI Plan Apo VC 60× water immersion objective (NA 1.2) was used for imaging. A 561 nm laser was employed for both excitation and photobleaching, and emission was collected using a 575–625 nm band-pass filter. Photobleaching was applied to 3–10 circular regions, 2 µm in diameter, each containing a single, isolated PML body. Laser power and scan speed during photobleaching were optimized to achieve substantial fluorescence reduction while maintaining sufficient signal to track the movement of partially bleached PML NBs. The beam power was set to 100 µW, and each circular region was scanned for 12.5 ms. Imaging was conducted at 1 frame per second with a 100-ms camera exposure. Pre-bleaching imaging consisted of 11 frames, followed by continuous imaging for >10 min to monitor fluorescence recovery. All experiments were performed on previously unexposed cells to avoid photodamage from prior illumination.

Fluorescence intensity traces were extracted using Nikon NIS-Elements AR Analysis software (version 5.42.02) and analyzed with custom-written scripts in MATLAB (R2024b, MathWorks). Fluorescence recovery was modeled using a single-exponential function:

$$I\left(t\right)=I_{0}+I_{\infty }\left(1-e^{t\;/\;\tau_{\mathrm{recovery}}}\right)$$

where $I\left(t\right)$ is the fluorescence intensity at time *t*, $I_{0}$ is the baseline intensity, $I_{\infty }$ is the recovered plateau intensity, and $\tau_{\mathrm{recovery}}$ is the characteristic recovery time. The nuclear localization of each PML NB was verified by differential interference contrast (DIC) brightfield and confocal microscopy.

### Structure and zinc coordination predictions using molecular dynamics simulations

The zinc-free and the two zinc-containing AlphaFold3-derived structures were prepared for molecular dynamics simulations using the Amber 22 program. Protein preparation was performed at pH = 7 with the Amber ff14SB force field, incorporating balancing Na^+^ counterions, explicit TIP3P water solvation, and NaCl at a concentration of 150 mM. Following system setup, all three structures underwent an identical regime of minimization, heating, and relaxation under constant pressure, with a stepwise decrease in the positional restraints of the atoms. In the final 20 ns of fully unrestrained relaxation, densities of 0.9959, 0.9952, and 0.9954 g/cm^3^ were recorded for the Zn-free, Zn_(1)_ and Zn_(2)_ structures, respectively. Lastly, the relaxed structures were submitted to a 200-ns production simulation at constant pressure (NPT ensemble) under periodic boundary conditions, employing a Langevin thermostat at 298 K and a timestep of 0.001 ps. All simulations were executed using the pmemd.CUDA module of Amber.

### Comparison of the organization of PML homologs between mouse and human

Exon-intron organization, domain architecture, signal sequences, and motif composition of individual PML protein isoforms, together with their amino acid sequence identity and length, were compared using EMBL-EBI Clustal Omega [48,49]. Analyses were based on previously published *PML* gene structures [34,36] and sequence data obtained from the NCBI GeneBank and UniProt repositories [38,39,50,51] (Mouse PML: mPML1, NM_008884.5; mPML2, NM_178087.4; mPML3, NM_001311088.1; mPMLX1, XM_006510860.3; mPMLX2, XM_006510861.3; mPMLX3, XM_006510862.5; mPMLX4, XM_006510864.5; mPMLX5, XM_030244128.2; mPMLX6, XM_017313212.3 and mPMLX7, PX092826, see Supplementary Figure S2. Human PML: hPMLI, NM_033238.3; hPMLII, NM_033239.3; hPMLIII, AAB19601.2, UniProt, P29590-9; hPMLIV, NM_002675.4; hPMLV, NM_033240.3; hPMLVI, NM_033244.4; hPMLVIIb, NM_033247.3).

## Results

### Endogenous expression of the confirmed, predicted, and novel murine PML isoforms

**Figure 1.**
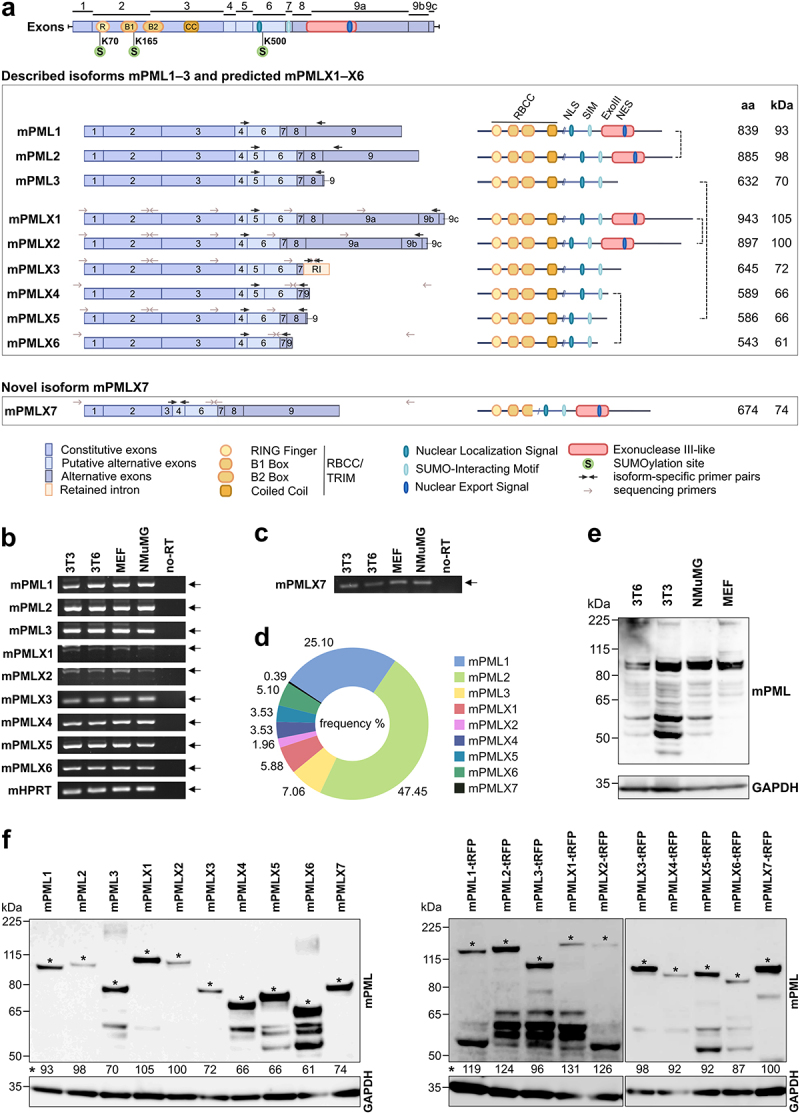
Expression of mPML isoforms. (a) Schematic representation of the exon and domain structure of the mPML isoforms. Upper panel: the alternative splicing of nine exons produces mRNA encoding nine individual mPML isoforms. The first three (mPML1 to 3) have been previously confirmed, while the other six (mPMLX1 to X6) were computationally predicted. All isoforms share the N-terminus sequence (exons 1–3) harboring the RBCC/TRIM domain consisting of a RING finger, two B-boxes and a coiled-coil (cc) domain. The dashed vertical lines (on the right) link isoforms that differ only in the presence or absence of exon 5. Alternative splicing of exons 7–9 produces unique C-termini for each isoform, with mPMLX3 uniquely retaining intronic sequence: mPML1 includes exons 4, 6–9; mPML2, 4–9; mPML3, 4–8 with a shortened exon 9; mPMLX1, 4–8 with a lengthened exon 9 containing two additional segments; mPMLX2, 4, 6–8 with the same extended exon 9; mPMLX3, 4–7 with retained intron; mPMLX4, 4–7 with a shortened exon 9 distinct from mPML3; mPMLX5, 4, 6–8 with a shortened exon 9 matching mPML3; mPMLX6, 4, 6, 7 with a shortened exon 9 matching mPMLX4; and mPMLX7, a uniquely shortened exon 3 with the remaining exons as in mPML2. All isoforms contain three major SUMOylation sites at positions K70, K165, and K500, a nuclear localization signal (NLS), and a SUMO-interacting motif (SIM). Additionally, isoforms mPML1 and 2 and X1 and X2 share a conserved exonuclease III-like (Exo III) domain. All domains, motifs, signal sequences, and specific sites were reproduced based on previous studies or predicted based on the homologous human PML system [27,34–36,52] (Supplementary figure S10b). Bottom panel: the newly discovered isoform mPMLX7 contains a uniquely shortened exon 3 that results in a six-amino acid C-terminal deletion in the B2 box and the complete loss of the cc domain. Black arrows represent the positions of primers used in this study to amplify the sequences of specific isoforms, whereas gray arrows denote those used for sequencing each isoform. b–d) Endogenous transcription of mPML isoforms. b–c) RT-PCR was performed on RNA isolated from the indicated cell lines using isoform-specific primer pairs (as indicated by black arrows in (a)). The *mHprt* reference gene was amplified as an internal control for cDNA integrity, and a no-reverse transcriptase (no-RT) control was included for each gene. (d) Semi-quantitative analysis of mPML isoform transcripts using cDNA nanopore sequencing. Schematic representation of the abundance of individual isoform transcripts. The number of reads corresponding to mRNAs encoding each isoform was normalized to the total number of reads (100%). (e) Protein expression of the endogenous mPML isoforms in various cell lines. Whole-cell lysates were analyzed by western blotting with anti-PML antibody. GAPDH served as a loading control. (f) Validation of mPML-tRFP/mPML-S constructs. Full-length mPML protein isoforms with (right panel) or without (left panel) the tRFP tag individually expressed in *Pml*^−/−^ MEFs. The cells were transfected with plasmids expressing individual isoforms, and cell lysates were analyzed by western blotting using an anti-PML antibody. GAPDH was visualized as a loading control. Isoform-specific molecular masses (MM) were calculated using the protein molecular weight tool in sequence manipulation suite [53]. The asterisk (*) indicates the approximate molecular mass of the mPML isoforms. Amino-acid (aa) lengths and predicted molecular masses of the isoforms are as follows: PML1, 839 aa, 93 kDa; PML2, 885 aa, 98 kDa; PML3, 632 aa, 70 kDa; PMLX1, 943 aa, 105 kDa; PMLX2, 897 aa, 100 kDa; PMLX3, 645 aa, 72 kDa; PMLX4, 589 aa, 66 kDa; PMLX5, 586 aa, 66 kDa; PMLX6, 543 aa, 61 kDa; and PMLX7, 674 aa, 74 kDa.

The NCBI database lists nine mPML isoforms generated by alternative splicing – three experimentally confirmed (mPML1 to 3) and six computationally predicted (mPMLX1 to X6) – for the murine *Pml* gene (Figure 1a) (NCBI, Gene ID: 18,854). To characterize the mPML system, we first examined whether the predicted isoforms are endogenously transcribed. We designed isoform-specific primer pairs (Supplementary Tab. S1) to perform reverse transcription-polymerase chain reaction (RT-PCR) using RNA prepared from various mouse cell lines and organs as a template. A schematic showing the primer binding sites (Figure 1a, black arrows), exon composition, and structural features of each isoform is presented in Figure 1a, upper panel. We detected specific PCR products corresponding to the predicted length for both previously confirmed and predicted mPML isoforms in all the tested samples. These comprised mouse embryo fibroblasts (3T3 cells, 3T6 cells, and mouse embryonic fibroblasts (MEFs)), epithelial cells derived from normal mammary gland tissue (NMuMG) (Figure 1b), and tissues from a C57BL/6NCrl mouse, including liver, spleen, kidney, lung, heart, brain, thymus, and muscle (Supplementary Figure S1). Quantification of the transcripts of the individual isoforms by RT-qPCR was not feasible due to the shared exon-exon junctions among the isoforms [27,37].

To further confirm the presence of full-length transcripts for each isoform, we performed RT-PCR on MEF-derived RNA, followed by Sanger sequencing (for specific primers, see Figure 1a, gray arrows and Supplementary Tab S2). We detected the full-length transcripts for all predicted isoforms (mPMLX1 to X6) except mPMLX3 (Supplementary Figure S2). For this isoform, although we detected the PCR amplicon of the expected size and we obtained only a partial sequence, including the N-terminal region common to all isoforms and a unique retained intronic sequence (nucleotides 1196–1740 missing; underlined in Supplementary Figure S2). Surprisingly, sequencing of the RT-PCR products from MEFs revealed a unique, previously undescribed mPML sequence containing an alternatively spliced exon 3. Notably, this isoform lacked the CC domain. A schematic showing the exonic composition and structural features of this putative isoform (named here mPMLX7, following the nomenclature assigned to the computationally predicted isoforms in the NCBI database [38,39]) is displayed in Figure 1a, bottom panel and its nucleotide sequence is depicted in Supplementary Figure S2 (NCBI accession number PX092826). The presence of a consensus splice site sequence within exon 3 (Supplementary Figure S3), suggested that this variant could represent another isoform of the mPML protein. Consequently, we designed primer pairs to amplify this transcript using the above-detailed experimental setup (Figure 1a, bottom panel, black arrows; Supplementary Table S1). We were able to confirm the endogenous transcription of this sequence in all tested cells and mouse organs (Figure 1c and Supplementary Figure S1).

To validate the PCR-based assay results and provide a semi-quantitative analysis of mPML isoform transcripts, we utilized a long-read third-generation sequencing technology, specifically cDNA Nanopore sequencing (Oxford Nanopore Technologies) [54–56], on RNA samples derived from MEFs. This sequencing approach employs a template-switching technique to enrich for full-length transcripts, which is essential for capturing the spectrum of mPML splice variants. Reads were successfully generated and aligned to the reference sequence of the potential novel isoform mPMLX7, as well as to all NCBI-predicted mPML isoforms except for mPMLX3. Figure 1d summarizes the relative abundance (percentages) of reads corresponding to reference transcripts encoding each isoform. Our results show that mPML variants 1 and 2 represent the most abundant isoforms, accounting for approximately 70% of total mPML reads. mPML3, mPMLX1 and mPMLX6 isoforms are present in similar percentages (approximately 6% each), while the remaining isoforms are the least abundant with percentages ranging from 0.39 to 3.53. In light of the above findings, we confirmed the newly discovered sequence mPMLX7 as a novel isoform of PML. For the following experiments, despite the important differences in expression of the isoforms in the mouse cells, we included all ten isoforms in subsequent characterization steps, including mPMLX3 as a probable but unconfirmed expressed isoform.

To follow the expression of the mPML isoforms at the protein level, we performed western blotting on lysates from the previously tested cell lines (Figure 1e). We detected multiple protein bands, ranging from 50 to 225 kDa, some of which corresponded to the predicted molecular masses of mPML protein isoforms while others likely represented their post-translationally modified (PTM) forms. The abundance of the individual bands varied among cell lines and cell types. Unfortunately, although the antibody specificity was confirmed using *Pml*^−/−^ MEFs (Supplementary Figure S4), precise identification of individual mPML isoforms with or without PTM solely via comparison of their molecular mass is not possible (Figure 1e). Therefore, we constructed expression plasmids for all the above-described mPML isoforms (mPML1 to X7) to verify their properties. Both untagged variants (mPML-S) and C-terminal turboRFP (tRFP)-tagged variants were generated to better characterize the mPML isoforms (Supplementary Table S3).

All the constructs were confirmed to produce the respective full-length proteins in both tagged and untagged variants. The plasmids were transfected into *Pml^−/−^* MEFs, and protein expression was analyzed using western blotting. All mPML isoforms were detected at the expected sizes, corresponding to their molecular masses (Figure 1f).

Altogether, our findings provide the first experimental evidence for the transcription of previously computationally predicted mPML isoforms, both *in vitro* and *in vivo*, as well as for the existence of a novel PML isoform, mPMLX7.

### Nuclear body formation by the individual murine PML isoforms

**Figure 2.**
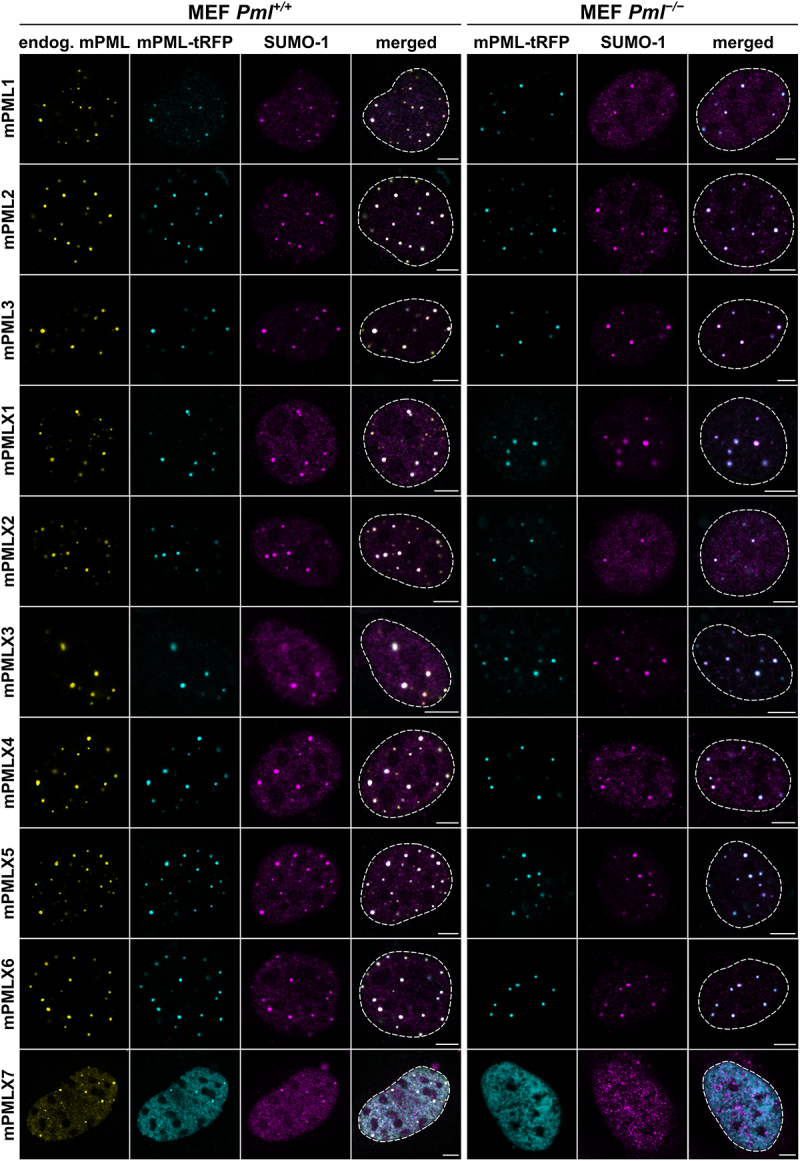
Formation of PML NBs for individual mPML isoforms. Confocal microscopy visualization of individual mPML isoforms expressed as fusion proteins with C-terminal tRFP tag (cyan) in *Pml^+/+^* or *Pml^−/−^* MEFs. Endogenous mPML proteins (yellow) and SUMO-1 proteins (magenta) were immunostained using specific antibodies. Cell nuclei were visualized by DAPI staining (borders of the nuclei indicated by a dashed line in the merge). Importantly, the anti-mPML antibody also detects the transiently expressed mPML-tRFP isoforms. Scale bar, 5 μm.

The ability of individual human PML isoforms to form functional NBs in both *Pml*^*+/+*^ and *Pml*^*−/−*^ conditions has been well established [37,57]. To assess this capacity in the murine PML system, we transiently expressed each mPML-tRFP isoform in both *Pml*^*+/+*^ and *Pml*^*−/−*^ MEFs. We simultaneously visualized mPML proteins along with SUMO-1, a key factor in both the assembly and disassembly of PML NBs, through immunostaining. Our results showed that all NCBI-listed mPML isoforms (mPML1 to X6) can form punctuate structures resembling PML NBs in *Pml*^*−/−*^ cells (Figure 2). SUMO-1 protein accumulation was evident in these NBs. In *Pml*^*+/+*^ cells, all exogenous mPML isoforms co-localized with endogenous mPML. Notably, the phenotype observed in both cell lines resembled that seen in non-transfected, *Pml*^*+/+*^ MEFs (Supplementary Figure S5). These results were further validated using mPML-S plasmids expressing mPML isoforms without the tRFP tag in *Pml*^*−/−*^ MEFs (data not shown).

Meanwhile, given that the novel isoform mPMLX7 lacks the CC domain, which is essential for PML multimerization [58], we did not expect it to aggregate into NBs. This assumption was confirmed in *Pml*^*−/−*^ MEFs, where mPMLX7 displayed a diffuse nuclear distribution. Surprisingly, mPMLX7 was able to incorporate into preexisting PML NBs when it was expressed in *Pml*^*+/+*^ MEFs (Figure 2).

Collectively, these results confirmed that all tested mPML isoforms can be incorporated into NBs in *Pml*^*+/+*^ MEFs, and that mPML1 through mPMLX6 are capable of forming NBs independently in a *Pml*^*−/−*^ background.

### Responses of individual murine PML isoforms to arsenic treatment

**Figure 3.**
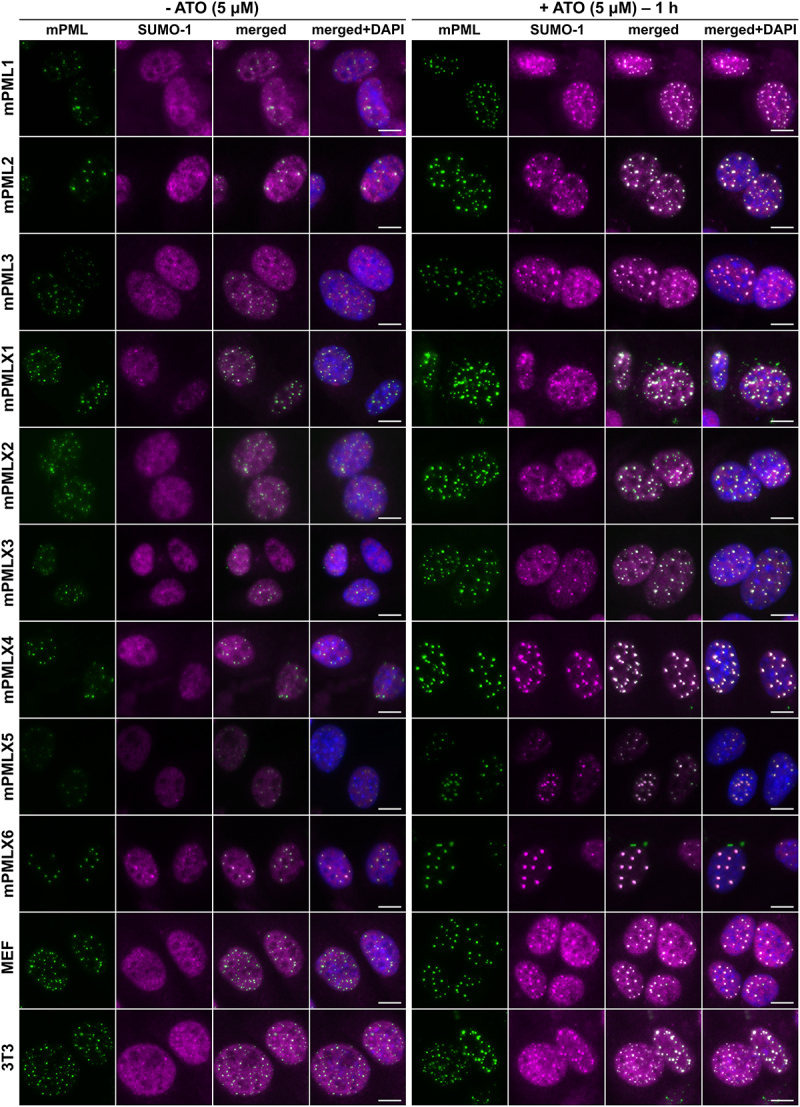
The effect of arsenic treatment on mPML NB dynamics. *Pml^−/−^* MEFs transiently expressing individual mPML isoforms or control *Pml^+/+^* MEFs or 3T3 cells were mock-treated or treated with 5 µM ATO for 1 h. Subsequently, the cells were fixed and stained using specific antibodies against mPML (green) and SUMO-1 (magenta) proteins. Cell nuclei were visualized by DAPI staining (blue). Representative fluorescence microscopy images are shown. Scale bar, 10 μm.

PML NBs have been shown to respond to stress stimuli including arsenic [59]. Accordingly, we hypothesized that analyzing the response of the nine isoform-specific NBs (mPML1 through mPMLX6) along with mPMLX7, which does not form NBs in *Pml*^*−/−*^ cells, to arsenic trioxide (ATO) could provide insights into their functionality. ATO induces rapid PML poly-SUMOylation, which is followed by the translocation of PML from the nucleoplasm to PML NBs and its subsequent ubiquitin-mediated proteasomal degradation [59–61]. To test the responses of individual mPML isoforms (non-tagged versions) to arsenic, each was transiently expressed in *Pml*^*−/−*^ MEFs, after which the cells were treated with ATO for 1, 6, or 24 h. Using fluorescent microscopy, we tracked the redistribution of SUMO and PML proteins within the PML NBs formed by each of the nine (mPML1 to X6) isoforms. After 1 h of ATO treatment, all the NBs exhibited increased accumulation of SUMO-1 and PML signals (Figure 3). At later time points, we observed either massive ‘aggregation’ of PML proteins mainly in cytosol or the absence of PML NBs for all nine isoforms (Supplementary Figures S6).

These findings were also confirmed in *Pml*^*+/+*^ MEFs and 3T3 cells not expressing exogenous PML. In these cells, the SUMO-1 and mPML signal intensities were increased after 1 h of ATO treatment (Figure 3b), while at later sampling times, the PML signal was decreased (Supplementary Figure S6).

Additionally, as expected, no mPMLX7 speckles were formed in response to ATO (Supplementary Figure S7).

**Figure 4.**
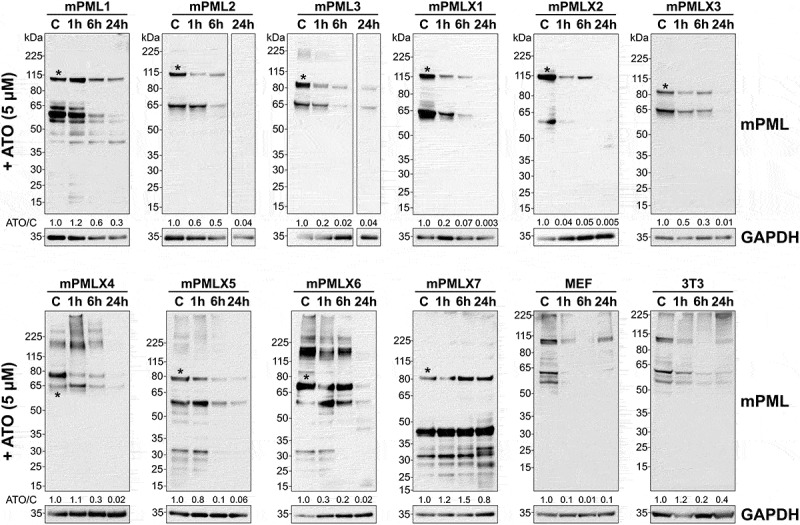
The effect of arsenic treatment on mPML protein levels in the cells. *Pml^−/−^* MEFs transiently expressing individual mPML isoforms or control *Pml^+/+^* MEFs or 3T3 cells were mock-treated (C) or treated with 5 µM ATO for the indicated times. Whole-cell lysates were analyzed by western blotting using an anti-PML or anti-GAPDH antibody. Asteriks (*) indicates the predicted molecular mass (MM) of the isoforms. ATO/C quantification represents the ratio of PML levels in ATO-treated cells (normalized to GAPDH) relative to untreated controls.

Furthermore, although previous studies have established that PML isoforms are typically highly SUMOylated, resulting in detection of high molecular weight (HMW) bands on Western blots [37,62], we were not able to detect any HMW bands efficiently in our analysis (Figure 1e). Despite this observation, we employed this method to monitor the overall levels of mPML proteins. We confirmed that except for mPMLX7, all PML protein isoforms (mPML1 to X6) underwent degradation following ATO treatment (Figure 4). While the levels of almost all isoforms were decreased after 1 h of treatment, prolonged ATO exposure (6 h) led to a reduction in the protein levels of all mPML isoforms, with protein levels nearly undetectable 24 h post-treatment. Unlike other isoforms, mPMLX7, which does not form NBs, was resistant to ATO treatment, potentially due to its inability to multimerize.

Finally, these findings were confirmed in *Pml*^*+/+*^ MEFs and 3T3 cells not expressing exogenous PML. After 6 h of exposure to ATO, western blotting showed that mPML protein was nearly undetectable (Figure 4).

Altogether, our data showed that mPML isoforms 1 to X6 respond to ATO treatment by undergoing degradation. Furthermore, the data suggests that this degradation mechanism involves the SUMOylation of mPML, similarly to their human counterparts, with mPMLX7 as a notable exception.

### Assembly dynamics of individual murine PML isoforms

**Figure 5.**
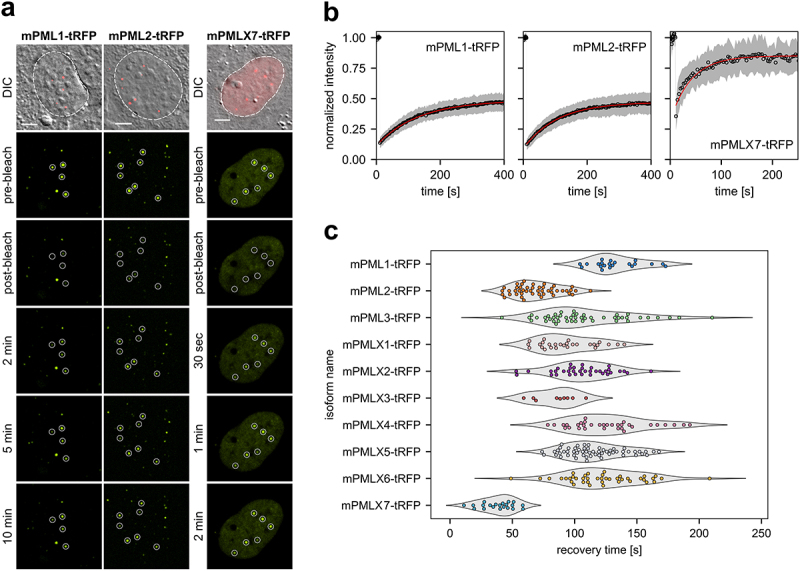
Exchange of individual mPML isoforms at endogenous PML NBs. (a) FRAP experiments were performed using mPML-tRFP constructs expressed in *Pml^+/+^* MEFs. Circular ROIs containing individual PML NBs were selected, and photobleaching was carried out by point-scanning following pre-bleach time-lapse imaging. Post-bleach fluorescence recovery was monitored over 10 min. Nuclear localization of PML NBs was confirmed via DIC microscopy. Representative isoforms are shown. Scale bar, 5 μm. (b) Representative isoform-averaged fluorescence recovery traces. Black circles indicate the mean across all recorded traces for the isoform, with the gray shaded area denoting ± one standard deviation. The red curve represents a single-exponential fit using the averaged parameters from individual fits. (c) Combined violin and swarm plot comparing recovery times across different isoforms.

The biogenesis of PML NBs predominantly relies on the scaffold function of the PML protein, facilitated by RBCC/TRIM-domain-driven self-assembly. As described in the human model, individual PML isoforms exhibit specific exchange kinetics at NBs, which underpins their unique properties [57,63]. To investigate the binding properties of PML in the endogenous murine model, we employed fluorescence recovery after photobleaching (FRAP) [57,63], a widely used approach for studying the dynamics of structures formed via liquid-liquid phase separation (LLPS) [62,64,65]. Single NBs formed by mPML-tRFP proteins incorporated into wild-type PML NBs were bleached to near-background levels in circular regions of interest (ROI), and the recovery of the fluorescent signal was monitored for at least 10 min (Figure 5a, representative isoforms). In line with observations from the human PML system, mPML isoforms 1 to X6 exhibited exchange at the NBs, with FRAP curves indicating the presence of slowly exchanging populations, with incomplete signal recovery within the observation period (Figure 5b, black circles, representative isoforms and Supplementary Figure S8). The recovery kinetics of individual mPML isoforms at NBs (Figure 5c) were determined via the single-exponential fitting of FRAP traces (Figure 5b, red curves, representative isoforms and Supplementary Figure S8). This model provided a good fit across all tested mPML isoforms, suggesting that the mPML isoforms were a single population of proteins with comparable exchange dynamics. In parallel with the human model, the mPML2 isoform, the murine homolog of hPMLI [62,63], showed the fastest recovery among mPML isoforms 1 to X6.

Intriguingly, mPMLX7 exhibited the fastest exchange rate, with near-complete fluorescence recovery observed after just 3 min. Given the diffuse nuclear localization of mPMLX7 proteins and their ability to incorporate only into preexisting mPML NBs, we hypothesized that their fast exchange rate reflected weak binding within the NB scaffold through RB1-mediated disulfide bridges, hydrophobic and electrostatic interactions, and specific interactions between the C-terminal domains. The observed fast exchange rate for mPMLX7 is in agreement with the previously characterized high mobility of PML proteins within the nucleoplasmic pool [19,63,64]. Collectively, these results demonstrated, that the ten mPML isoforms undergo dynamical exchange at endogenous mPML NBs, further supporting their ability to form functional structures.

### Analysis of the 3D structures of murine PML isoforms using AlphaFold3

**Figure 6.**
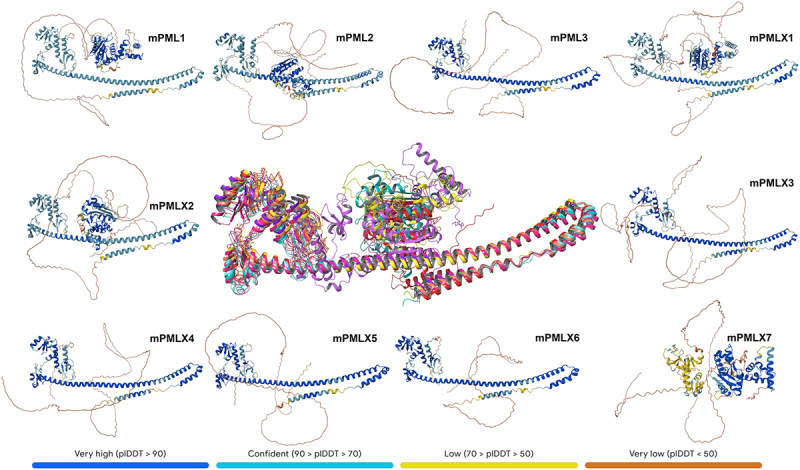
AlphaFold3 structural prediction for isoforms of mPML. Full structures are given individually and are overlayed in the central image, with low-confidence regions hidden (residues 1–49 and 403–570). Additional hidden residues are 766–885 for mPML2; 867–943 form mPMLX1; 821–897 for mPMLX2; and 239–408 for mPMLX7. Confidence scores per residue, based on predicted local distance difference test (pLDDT) values, are provided by AlphaFold and color-coded as described in the legend.

The predicted structure of major human PML isoforms was recently published, with a complete alignment observed in their common N-terminal RBCC regions [64]. To determine the 3D structures of the individual mouse PML isoforms, we analyzed their sequences using AlphaFold3 software. As expected, all mPML isoforms exhibited identical folding in their conserved RBCC domain, except for the novel isoform mPMLX7, which lacks the six-amino-acid C-terminal part of the B2 box and the entire CC domain (Figure 6).

### Zinc coordination by the novel murine mPMLX7 isoform

**Figure 7.**
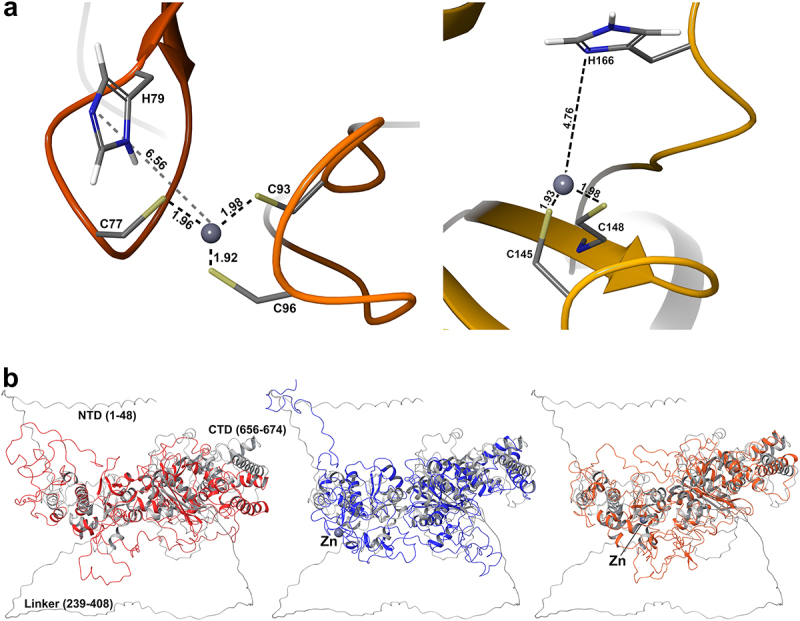
MD simulation results of mPMLX7. (a) Coordination of the Zn^2+^ cation in the two AlphaFill-suggested positions Zn_(1)_ (left) and Zn_(2)_ (right). The structures were extracted from the last frame of their respective production runs. (b) Superposition of the post-MD (left) Zn-free (red), (middle) Zn_(1)_ (blue), and (right) Zn_(2)_ (orange) structures over the AlphaFold prediction (gray). Left: the disordered regions have been labeled on the AlphaFold prediction. Middle-right: the Zn^2+^ positions are labeled and shown with blue balls.

The RBCC domain of the human PML protein harbors three zinc-binding domains – RING finger, B1 box and B2 box – each coordinating two zinc cations [66–69]. The integrity of the zinc binding residues is crucial for correct PML NB formation, as mutations in these ligands disrupt PML NB biogenesis [28,68,70,71]. The novel mPMLX7 isoform harbors a unique deletion within its RBCC domain that results in the loss of the CC domain and six amino acids at the B2 box in the C-terminus. To test the zinc-binding potential and structural stability of mPMLX7, its sequence was submitted to the AlphaFold Server [72] for structure prediction. Out of five proposed structures, the one with the best ranking scores (0.62) and the lowest disorder fraction (0.34) was chosen for molecular dynamics (MD) simulation. Subsequently, the prediction of the location of the Zn^2+^ cation in the PML protein was obtained from the AlphaFill databank (UniProt code Q60953) [73]. Four sites were proposed by AlphaFill (https://alphafill.eu/model?id=Q60953), with two being flagged as suspect. Hence, only the two most favorable sites were chosen for further analysis (Figure 7a). One, referred to as Zn_(1)_ encompassed cysteines 77C, 93C, and 96C, while the other, termed Zn_(2)_, involved 145C and 148C. Both were located in exon 2.

The mPMLX7 structure provided by AlphaFold comprises two structured regions encompassing residues 49–238 and 409–655, interspersed by the disordered N- and C- terminal domains (residues 1–48 and 656–674, respectively), and containing an elongated linker (239–408) connecting the two structured regions (Figure 7b, left). To assess their stability (see Materials and Methods), 200-ns production runs were performed on the three predicted structures, with substantial rearrangement of the disordered domains being observed after each of the three runs (Supplementary Figure S9). The post-MD structures, overlayed on the original prediction in grayscale, are shown in Figure 7b. Cα RMSD values of the structured regions between the initial AlphaFold model and the last MD frame were 8.2, 6.4 and 5.9 Å for Zn-free, Zn_(1)_, and Zn_(2)_, respectively. When disordered regions were included, RMSD values exceeded 19 Å for all three structures. These MD results attest to a degree of stability in the novel variant. Moreover, the results of the simulations for the Zn-containing structures supported that mPMLX7 retains partial function integrity.

## Discussion

Over the past three decades, substantial progress has been made in research on the PML protein and PML NBs based on gene silencing and knockout studies. Nevertheless, the mechanisms underlying their biogenesis and functions remain largely elusive. As membraneless organelles, PML NBs are extremely versatile structures that orchestrate the sequestration of partner proteins, mediate their post-translational modifications, and facilitate specific interactions, thereby achieving a diverse range of crucial cellular functions, from genome maintenance to tumor suppression [7,16,74–76].

As individual PML isoforms have been identified and characterized, limitations in studying the functions of PML NBs have become increasingly apparent. Current approaches primarily rely on the expression of individual human isoforms in *PML*^*−/−*^ cells, with HeLa *PML*^*−/−*^ cells serving as the primary model system. However, their oncogenic origin presents significant challenges for interpreting isoform-specific functions in physiological contexts, given that these cells exhibit compromised tumor suppressor and stress response pathways, altered SUMOylation, and irregular nuclear morphology [77–82]. *Pml*^*−/−*^ mouse embryonic fibroblasts present an alternative model to HeLa cells. However, emerging evidence indicates that the behavior of human PML isoforms differs between mouse and human models [57], raising concerns about the generalizability of research findings. This highlights the need for a deeper understanding of the composition and functions of PML NBs in the mouse model, not only to improve the translatability of mouse-to-human research but also because mouse models remain a cornerstone of scientific investigation.

Like human *PML*, *mPml* undergoes alternative 3’-terminal splicing of nine exons, producing mRNAs encoding isoforms that have an identical N-terminal structure but differ significantly in the composition of their C-terminus. To date, only three isoforms have been described for murine PML (mPML1 to 3) [36–39,50]. Here, we confirmed for the first time the endogenous expression of five out of six additional isoforms that were previously only computationally predicted (mPMLX1, mPMLX2 and mPMLX4 to mPMLX6), as well as one novel isoform, mPMLX7. This highlights the marked capacity of the *PML* gene to produce diverse variants through alternative splicing. Long-read transcriptome analysis of the *mPml* gene revealed a differential abundance of individual mouse PML isoforms. Specifically, variants 2 and 1 were found to be most highly transcribed isoforms relative to the other variants identified here.

In this study we were unable to detect the full-length transcript sequence of one predicted variant, mPMLX3. Therefore, additional studies are required to demonstrate whether its expression could be substantial, for example in response to specific stimuli.

Furthermore, given the limitations in detecting individual mPML protein isoforms in cells, we were unable to determine precisely their endogenous translation. To address this issue, we established a new model system for PML research, comprising plasmids encoding ten mPML isoforms, that can be individually expressed in *Pml*^*−/−*^ MEFs. We acknowledge that a constraint of our study is the lack of suitable antibodies or methods to study the translation of specific isoforms endogenously; therefore, our analysis relies on the overexpression system. This issue highlights an urgent need to develop methodologies capable of overcoming the difficulties concerning the low-abundance proteins and the small differences in molecular mass.

Until now, only the mPML2 and hPMLI isoforms have been considered as a homologous pair [36], with no clear counterparts identified for other isoforms. Interestingly, the exon-intron and predicted domains analysis (Supplementary Figure S10) reveals that mPMLX3 shares the same composition with hPMLV. Nevertheless, the exchange kinetics of mPMLX3 at endogenous NBs observed in our study did not correspond to those reported for the hPMLV [63]. Moreover, considering the limitations to detect the levels of transcription and translation of this isoform in mouse cells, further studies will be required for its validation and understanding the potential homology with hPMLV.

Interestingly, the splicing of exons 4–6 has been suggested to give rise to three ‘a–c’ subtypes of each isoform (‘a’: loss of exon 4; ‘b’: loss of exons 5 and 6; and ‘c’: loss of the three exons) [31], which is in agreement with identification of hPMLIa, hPMLIIa, hPMLIVa or hPMLVIb. In the murine PML system, this concept is further supported by the observation that three isoform pairs – mPML1/2, mPML3/X5 and mPMLX1/X2 – differ only by the inclusion or exclusion of exon 5 (subtype ‘a’).

In addition to isoform variation described above, exons 7 and 8 of human PML are further subdivided into their ‘a’ and ‘b’ subparts. While exons 7a and 8a correspond to murine exons 7 and 8, no equivalents of the human ‘b’ subparts have been identified in murine PML [27,34,37]. Notably, hPML exon 8b encodes a unique 13-amino-acid sequence specific to the hPMLIV isoform, which is essential for its pro-senescence function. Within this sequence, three bulky hydrophobic/cyclic residues (F623, Y627, and F631) at the C-terminus were shown to trigger p53-driven senescence. Intriguingly, the corresponding mouse *Pml* gene sequence, if spliced analogously, would encode for three large hydrophobic residues (Supplementary Figure S11) [37,83]. Further studies on this potential mouse isoform are required, for example, in conditions where cells are stimulated to enter senescence.

In this study, we identified a novel isoform, mPMLX7, characterized by a unique splicing event within the conserved RBCC domain in exon 3, resulting in the loss of the CC domain and six amino acids at the B2 box terminus. This ‘aberrant’ GT splice site although absent in *hPML*, is present in several PML orthologs across diverse taxonomic groups, including birds, rodents, whales, and even-toed ungulates, notably, *Sus scrofa* as demonstrated by Basic Local Alignment Search Tool (BLAST) analysis [84–86].

PML NBs are dynamic structures involved in the regulation of sorting and trafficking of partner proteins. Strikingly, PML isoforms were shown to exchange between NBs and nucleoplasm. Given the high mobility of PML outside NBs, it has been proposed that PML scans the nuclear volume for binding new partners or to seed new structures [19,63,64]. Here, we showed that nine mPML isoforms (mPML1 to X6) also underwent dynamic turnover at endogenous NBs, exhibiting isoform-specific exchange rates, as occurs in the hPML system. The fastest turnover was observed for isoform mPML2, consistent with its hPMLI homology. Conversely, none of the mPML isoforms exhibited the exceptionally slow exchange rate observed for hPMLV [63].

PML NB biogenesis greatly relies on SUMOylation, making it the best-studied PML posttranslational modification [34,57,87]. Notably, poly-SUMOylation and the SIM motif are required for efficient arsenic-induced PML proteasomal degradation [61,88–90].

ATO treatment induces PML cross-linking via disulfide bridges, PML oligomerization, and NB formation, resulting in increased interaction with SUMO-conjugating enzyme UBC9 and, eventually poly-SUMOylation [91].

In this study, we examined the responses of the individual PML isoforms to arsenic exposure. We noted that all isoforms from mPML1 to mPMLX6 were degraded with arsenic treatment, which was expected given the conservation of the SIM motif in all these isoforms. Soon after ATO exposure, the extent of SUMO-1 modification increased, as shown by immunostaining. We could not, however, demonstrate this by western blotting. This discrepancy may be due to deSUMOylation likely occurring *ex vivo* during cell lysis and sample preparation, despite the inclusion of N-Ethylmaleimide to inhibit SUMO-specific proteases.

As anticipated, the novel isoform mPMLX7 exhibited behavior distinct from that of the other nine isoforms (mPML1 to X6), and did not undergo degradation following ATO treatment or participate in the formation of punctuate structures in *PML*^*−/−*^ MEFs. Based on current knowledge of PML, we attribute this difference in behavior to the loss of the CC domain. Until recently, the accepted model for PML NB biogenesis was thought to involve a series of sequential steps. In this model, LLPS pre-condensation driven by weak interactions between the intrinsically disordered C-terminal regions of PML proteins initially provides the necessary concentration for the oxidation-dependent formation of disulfide bridges, along with electrostatic and hydrophobic interactions between the RING-finger and B1 box domains. Next, aggregated PML proteins undergo SUMOylation, further multimerization, and the recruitment of partner proteins via SUMO/SIM contacts [64,92]. However, recent studies suggest an alternative mechanism in which the RING-finger, B1 box and B2 box domains of PML monomers are folded around an α3 helix and are displayed on the outer surface of these molecules. This configuration allows the B2 box to be targeted by arsenic, thereby initiating the RING domain-dependent recruitment of UBC9. PML monomers then dimerize through RBCC interactions and further aggregate via a unique antiparallel octopus-like CC domain assembly of neighboring PML dimers, leading to LLPS separation, polymerization, and SUMO/SIM-mediated partner recruitment [58]. Thus, the resistance of mPMLX7 to arsenic treatment supports the alternative model of PML NB formation.

Furthermore, ATO was recently described to bind inside a C213 tri-cysteine pocket formed by a B2 trimer, driving a gel-like transition of PML NBs [93]. Additionally, higher-order RING domain assembly is crucial for efficient UBC9 recruitment [94]. As the mPMLX7 C217 residue (which correspond to the C213 residue in the human PML protein) is retained in exon 3, we hypothesize that its inability to assemble into higher-order NBs, and not ATO binding, is what prevents ATO-induced degradation.

Meanwhile, proper PML NB formation is dependent on the ability of the RING and B-box domains to coordinate zinc cations [95,96]. Using AlphaFold and AlphaFill analyses, we identified four candidate Zn^2+^ binding sites in the mPMLX7 sequence, all corresponding to sites in the RING and B1 domains of hPML, with two of them showing higher confidence scores [68,95]. Although mPMLX7 fails to form aggregates in *PML*^*−/−*^ cells, we propose that it may weakly bind to preexisting NBs via specific RB-driven interactions and interactions of C-terminal unstructured domains, possibly explaining its high exchange recovery rates upon bleaching [58,63,64].

Although a clear distinction has not yet been established between the functions of PML NBs and the PML protein itself, the higher-order structural and functional aggregates that form NBs represent only a minor fraction of total nuclear PML [59]. Most PML proteins are dispersed throughout the nucleoplasm, and their functions are largely unknown. The apparent inability of mPMLX7 to assemble into NBs – likely due to its aberrant splice site – may render it as a good candidate for studying the poorly understood roles of dispersed nucleoplasmic PML, particularly given that it has an intact C-terminus corresponding to exons 4–9.

Overall, the murine PML isoform system introduced here represents a model that can be used not only for basic research aimed at understanding the isoform-specific roles of PML in regulating the structure and functions of PML NBs, as emerging evidence highlights distinct functions of individual PML isoforms [13,17,34]. It can also be used to improve the translatability of findings from mouse to human systems.

## Supplementary Material

Anderova et al 2025 Supplement_FINAL tracked changes.docx

## Data Availability

The data that support the findings of this study are available upon reasonable request. Sequencing data generated in this study for the PML isoform mPMLX7 has been submitted to NCBI GenBank under accession number (PX092826). Raw transcriptome data are published under NCBI BioProject #PRJNA1381475, BioSample #SAMN54106409 as raw sequences in the Sequence Read Archive #SUB15828003.
