# Supplementary material for: Discovery of novel murine PML isoforms
Source: Nucleus. 2026 Mar 23;17(1):2646815. doi: 10.1080/19491034.2026.2646815 (PMC13011596; doi:10.1080/19491034.2026.2646815)
Supplement: Anderova et al 2025 Supplement_FINAL tracked changes.docx [file KNCL_A_2646815_SM4621.docx]

**Supplementary material**

**Table S1.** Isoform-specific primer pairs used for RT-PCR detection, including their annealing temperatures and corresponding amplicon lengths.

**Table S1 alt text:** Table summarizing primers used for RT-PCR detection of individual murine PML isoforms and the control murine hypoxanthine phosphoribosyltransferase gene, including primer sequences (5’–3’), annealing temperatures (°C), and amplicon lengths (base pairs).

| **Gene / Isoform** | **Primers** | **Sequence (5’-3’)** | **Annealing temperature** | **Amplicon (bp)** |
| --- | --- | --- | --- | --- |
| mPML1 | mPML1, X2, X5, X6-F  mPML1,2-R | ACCTTGAGCAGGAGGCTT  AGCACACGGAACTTGCTTT | 63 °C | 529 |
| mPML2 | mPML2, 3, X1, X4-F  mPML1,2-R | CACCTACAGAGAGGAGGCTTC  AGCACACGGAACTTGCTTT | 63 °C | 531 |
| mPML3 | mPML2, 3, X1, X4-F  mPML3, X5-R | CACCTACAGAGAGGAGGCTTC  CTAATTTTTTTCATTGTCAATCTTG | 58 °C | 484 |
| mPMLX1 | mPML2, 3, X1, X4-F  mPMLX1, X2-R | CACCTACAGAGAGGAGGCTTC  CACCATGAATCGAACATCCAG | 62 °C | 1386 |
| mPMLX2 | mPML1, X2, X5, X6-F  mPMLX1, X2-R | ACCTTGAGCAGGAGGCTT  CACCATGAATCGAACATCCAG | 62 °C | 1384 |
| mPMLX3 | mPMLX3-F  mPMLX3-R | AGCTTCATCCTGACCCTGAC  AGGTAGCAGATGCTGACAATTC | 63 °C | 104 |
| mPMLX4 | mPML2, 3, X1, X4-F  mPMLX4, X6-R | CACCTACAGAGAGGAGGCTTC  TTCTGGGCAGATTCTCGGT | 64 °C | 332 |
| mPMLX5 | mPML1, X2, X5, X6-F  mPML3, X5-R | ACCTTGAGCAGGAGGCTT  CTAATTTTTTTCATTGTCAATCTTG | 58 °C | 482 |
| mPMLX6 | mPML1, X2, X5, X6-F  mPMLX4, X6-R | ACCTTGAGCAGGAGGCTT  TTCTGGGCAGATTCTCGGT | 64 °C | 330 |
| mPMLX~~K~~7 | mPMLX~~K~~7-F  mPMLX~~K~~7-R | TCTCCATTGCGATATTGATGCAG  GAAGCCTCCTGCTCAAGGT | 64 °C | 120 |
| mHPRT | mHPRT-F  mHPRT-R | AGGACCTCTCGAAGTGTTGG  TTGCAGATTCAACTTGCGCT | 64 °C | 183 |

**Table S2.** Primers used for detection of full-length transcripts of the previously predicted mPMLX1 to X6 isoforms and the novel isoform mPMLX~~K~~7.

**Table S2 alt text:** Table summarizing primers used for detection of full-length murine PML transcripts, including primer sequences (5’–3’) and reaction temperatures (°C; incubation, annealing, and melting). Primers used for cDNA synthesis, PCR, and Sanger sequencing are listed.

| ***cDNA synthesis*** | | | |
| --- | --- | --- | --- |
| **Primer** | **Sequence (5’-3’)** | **Isoform** | **Incubation** |
| mPMLX1, X2-R1 | TCAAAGCTGGAACTCTGGGCTATC | mPMLX1, X2 | 50 °C |
| mPMLX3-R | CAAATGGGTAGAGGGTGTAG | mPMLX3 | 50 °C |
| mPMLX4, X6, X~~K~~7-R | CTAGGCCAGGCATCCCTTACTTT | mPMLX4, X6, X~~K~~7 | 42 °C |
| mPMLX5-R | CTAATTTTTTTCATTGTCAATCTTG | mPMLX5 | 50 °C |
| ***PCR*** | | | |
| **Primer** | **Sequence (5’-3’)** | **Isoform** | **Annealing temperature** |
| mPMLX1, X2, X3-F  mPMLX1, X2-R2 | GCCTTCACAGACCTAATCAAGAAATGG  ATCTGGAAAACACCATGAATCGAACATC | mPMLX1, X2 | 67 °C |
| mPMLX1, X2, X3-F  mPMLX3-R | GCCTTCACAGACCTAATCAAGAAATGG  TAGAGTGAGCCTCCTCACAGG | mPMLX3 | 65 °C |
| mPMLX4, X6, X~~K~~7-F  mPMLX4, X6, X~~K~~7-R | CACCATGGAAACTGAACCAGTTTCCGT  CTAGGCCAGGCATCCCTTACTTT | mPMLX4, X6, X~~K~~7 | 67 °C |
| mPMLX5-F  mPMLX5-R | AGACCTAATCAAGAAATGGA CTAATTTTTTTCATTGTCAATCTTG | mPMLX5 | 57 °C |
| ***Sanger sequencing*** | | | |
| **Primer** | **Sequence (5’-3’)** | **Isoform** | **Melting temperature** |
| mPMLX1, X2, X3-F | GCCTTCACAGACCTAATCAAGAAATGG | mPMLX1, X2, X3 | 58 °C |
| mPMLX5-F | AGACCTAATCAAGAAATGGA | mPMLX5 | 48 °C |
| SEQ Exon2-F | TTCAGTGAGCAGCTTCCTCG | mPMLX1, X2, X3, X5 | 57 °C |
| SEQ Exon2-R | GAAGATATTGGACTTGCGCGTACTG | mPMLX1, X2, X3, X5 | 58 °C |
| SEQ Exon3-F | CTCTGTAGCTTGCGCCAGG | mPMLX1, X2, X3, X5 | 58 °C |
| SEQ Exon6-F1 | TCCAACCCTGAGAGCACCG | mPMLX1, X2, X3, X5 | 60 °C |
| SEQ Exon6-F2 | GAGGAAGTGCTCCCACGAAG | mPMLX4, X6, X~~K~~7 | 55 °C |
| SEQ Exon6/7-F | CAACTCGCTCCTCTGTTTCC | mPMLX4, X6, X~~K~~7 | 55 °C |
| SEQ Exon9-F | CCCTCATCCGGGAACGCATA | mPMLX1, X2 | 59 °C |
| SEQ X3intron-F | AGCTTCATCCTGACCCTGAC | mPMLX3 | 57 °C |
| SEQ X3intron-R | AGGTAGCAGATGCTGACAATTC | mPMLX3 | 55 °C |
| M13-F | GTAAAACGACGGCCAGT | mPMLX4, X6, X~~K~~7 | 50 °C |
| M13-R | AACAGCTATGACCATG | mPMLX4, X6, X~~K~~7 | 50 °C |

**
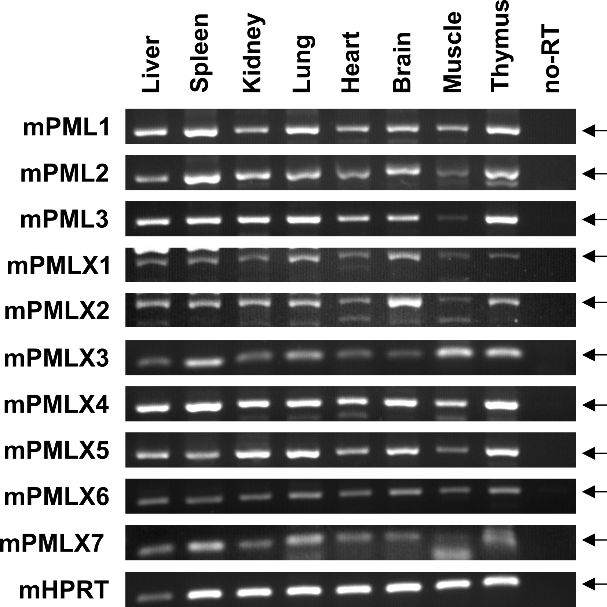
**

**Figure S1.** Endogenous transcription of mPML isoforms in various mouse organs. RT-PCR was performed on RNA isolated from the indicated tissues using isoform-specific primer pairs. The *mHrpt* reference gene was amplified as an internal control for cDNA integrity, and a no-reverse transcriptase (no-RT) control was included for each gene.

**Figure S1 alt text:** Agarose gel images showing RT-PCR amplification products of individual murine PML isoform transcripts from multiple mouse organs, including liver, spleen, kidney, lung, heart, brain, muscle and thymus.

>NM_008884.6:44-2563 Mus musculus promyelocytic leukemia (Pml), transcript variant 1, mRNA

ATGGAAACTGAACCAGTTTCCGTGCAGAAGGTACCTGCACCCCCTGGATCTCCCTGTCGACAACAGGACTCTGCCCTGACCCCCACGCCCACCATGCCTCCCCCAGAGGAACCCTCCGAAGACTATGAACACAGCCAAAGCCCTGCAGAGCAAGCCATACAGGAGGAATTTCAGTTTCTGCGCTGCCCGAGCTGCCAGGCCCAAGCCAAGTGCCCCAAACTGCTGCCTTGCCTGCACACGCTGTGCTCCGGATGCCTGGAGGCGCCTGGCCTGCAGTGCCCCATCTGCAAGGCTCCTGGGCAGGCCGATGCTAATGGGGAGGCCCTGGATAACGTGTTCTTCGAGAGCCTGCAGCGGCGCCTGGCGGTGTTCCGGCAGATCGTGGATGCTCAGGCTGCGTGCACCCGCTGCAAAGGCTTGGCCGACTTCTGGTGTTTCGAGTGTGAACAGCTCATTTGCAGCAAGTGCTTCGAAGCACACCAGTGGTACCTCAAGCATGAGGCCCGGCCCCTGGCTGATCTCCGCGACAATTCAGTGAGCAGCTTCCTCGACAGTACGCGCAAGTCCAATATCTTCTGCTCCAATACCAACCACCGCAACCCTGCGCTGACTGACATCTACTGCCGAGGCTGCGCCAAGCCTCTGTGTTGCACATGCGCGCTCCTGGACCGCAACCACAGCCATCTCCATTGCGATATTGGTGAGGAGATTCAGCAGTGGCATGAGGAACTAGGCACCATGACACAGACTCTGGAGGAGCAGGGCAGAACCTTCGACAGTGCCCATGCACAGATGTGCTCAGCTATAGGACAGCTGGACCACGCACGCGCAGACATTGAGAAGCAGATCCGCGCACGCGTGCGCCAGGTGGTAGACTACGTGCAGGCTCAGGAGCGCGAGCTGCTCGAGGCGGTGAATGACCGCTACCAGCGCGACTACCAGGAAATAGCTGGCCAGCTGAGCTGCCTGGAAGCTGTGCTGCAGCGCATCCGCACTAGTGGGGCGCTGGTCAAGAGGATGAAGCTCTATGCCTCCGACCAGGAGGTGCTGGATATGCACAGCTTTCTGCGCAAGGCACTCTGTAGCTTGCGCCAGGAGGAGCCCCAGAACCAGAAAGTCCAGCTGCTCACCAGAGGTTTCGAGGAGTTCAAGCTGTGCCTGCAGGACTTCATCTCCTGCATCACCCAGAGGATAAATGCAGCTGTAGCCAGCCCAGAGGCAGCCAGCAATCAACCAGAGGCAGCCAGCACTCACCCAGTGACAACCAGCACGCCTGAGGACCTTGAGCAGGAGGCTTCTCAGACAGTCGGCTCCATGAAGAGGAAGTGCTCCCACGAAGATTGCTCCAGGAAGATCATCAAGATGGAGTCCACAGAGGAGAACGAGGACAGGTTGGCCACAAGCTCCCCCGAGCAGTCCTGGCCCAGCACTTTCAAGGCCACCTCCCCTCCCCATCTGGATGGGACTTCCAACCCTGAGAGCACCGTCCCTGAAAAAAAGATCCTCCTGCCCAACAACAATCATGTTACCAGTGACACAGGGGAAACAGAGGAGCGAGTTGTGGTGATCAGCAGCTCAGAGGATTCGGACACCGAGAATCTGTCCTCCCACGAGTTGGACGATAGCAGCAGTGAGTCCAGCAGCCTGCAGCTGGAGGGCCCCAATTCCCTCAAGGCCTTGGATGAGAGCCTCGCCGAACCCCACTTAGAAGACAGGACCCTGGTTTTCTTTGACCTCAAGATTGACAATGAAACCCAGAAAATTAGCCAGCTGGCCGCGGTGAACCGGGAAAGCAAGTTCCGTGTGCTCATCCAGCCCGAGGCCTTCAGTGTCTACTCCAAAGCTGTCTCCCTGGAGGCGGGGCTCCGGCACTTCCTCAGCTTCCTCACCACCATGCACCGTCCCATCTTGGCGTGTTCTAGGCTGTGGGGGCCGGGACTCCCCATCTTCTTCCAGACCCTGAGTGATATTAACAAGCTGTGGGAATTCCAGGACACCATCTCAGGTTTCTTGGCCGTGCTGCCCCTCATCCGGGAACGCATACCCGGCGCTAGCAGCTTCAAACTTGGGAACCTAGCCAAGACCTACCTGGCGAGAAACATGAGCGAACGCAGCGCTCTGGCTTCTGTGCTGGCCATGAGGGACTTGTGCTGCCTCCTTGAGATCTCCCCAGGACTGCCGCTGGCCCAGCATATCTACTCCTTTAGTAGCTTGCAGTGTTTTGCTTCCCTGCAGCCCCTAATTCAGGCCAGCGTCCTGCCACAGTCCGAGGCCCGCCTCTTGGCCCTCCACAATGTGAGCTTTGTAGAGTTACTGAATGCATATCGCACCAACAGGCAAGAAGGCTTGAAGAAGTATGTCCACTATCTGAGCCTGCAGACCACCCCGTTGTCATCGTCGGCTTCCACCCAAGTTGCCCAATTCCTGCAGGCTCTTAGCACCCACATGGAAGGACTGTTGGAAGGCCATGCCCCTGCTGGGGCAGAAGGCAAAGCTGAAAGTAAGGGATGCCTGGCCTAG

>NM_178087.5:44-2701 Mus musculus promyelocytic leukemia (Pml), transcript variant 2, mRNA

ATGGAAACTGAACCAGTTTCCGTGCAGAAGGTACCTGCACCCCCTGGATCTCCCTGTCGACAACAGGACTCTGCCCTGACCCCCACGCCCACCATGCCTCCCCCAGAGGAACCCTCCGAAGACTATGAACACAGCCAAAGCCCTGCAGAGCAAGCCATACAGGAGGAATTTCAGTTTCTGCGCTGCCCGAGCTGCCAGGCCCAAGCCAAGTGCCCCAAACTGCTGCCTTGCCTGCACACGCTGTGCTCCGGATGCCTGGAGGCGCCTGGCCTGCAGTGCCCCATCTGCAAGGCTCCTGGGCAGGCCGATGCTAATGGGGAGGCCCTGGATAACGTGTTCTTCGAGAGCCTGCAGCGGCGCCTGGCGGTGTTCCGGCAGATCGTGGATGCTCAGGCTGCGTGCACCCGCTGCAAAGGCTTGGCCGACTTCTGGTGTTTCGAGTGTGAACAGCTCATTTGCAGCAAGTGCTTCGAAGCACACCAGTGGTACCTCAAGCATGAGGCCCGGCCCCTGGCTGATCTCCGCGACAATTCAGTGAGCAGCTTCCTCGACAGTACGCGCAAGTCCAATATCTTCTGCTCCAATACCAACCACCGCAACCCTGCGCTGACTGACATCTACTGCCGAGGCTGCGCCAAGCCTCTGTGTTGCACATGCGCGCTCCTGGACCGCAACCACAGCCATCTCCATTGCGATATTGGTGAGGAGATTCAGCAGTGGCATGAGGAACTAGGCACCATGACACAGACTCTGGAGGAGCAGGGCAGAACCTTCGACAGTGCCCATGCACAGATGTGCTCAGCTATAGGACAGCTGGACCACGCACGCGCAGACATTGAGAAGCAGATCCGCGCACGCGTGCGCCAGGTGGTAGACTACGTGCAGGCTCAGGAGCGCGAGCTGCTCGAGGCGGTGAATGACCGCTACCAGCGCGACTACCAGGAAATAGCTGGCCAGCTGAGCTGCCTGGAAGCTGTGCTGCAGCGCATCCGCACTAGTGGGGCGCTGGTCAAGAGGATGAAGCTCTATGCCTCCGACCAGGAGGTGCTGGATATGCACAGCTTTCTGCGCAAGGCACTCTGTAGCTTGCGCCAGGAGGAGCCCCAGAACCAGAAAGTCCAGCTGCTCACCAGAGGTTTCGAGGAGTTCAAGCTGTGCCTGCAGGACTTCATCTCCTGCATCACCCAGAGGATAAATGCAGCTGTAGCCAGCCCAGAGGCAGCCAGCAATCAACCAGAGGCAGCCAGCACTCACCCAGTGACAACCAGCACGCCTGAGGACCTTGAGCAGCCCAAAGAAGTGCAGAGTGTACAGGCTCAGGCCCTAGAGCTGTCTAAGACCCAACCTGTGGCTATGGTAAAAACAGTGCCTGGAGCACACCCTGTACCAGTGTATGCCTTTTCAATGCAAGGCCCCACCTACAGAGAGGAGGCTTCTCAGACAGTCGGCTCCATGAAGAGGAAGTGCTCCCACGAAGATTGCTCCAGGAAGATCATCAAGATGGAGTCCACAGAGGAGAACGAGGACAGGTTGGCCACAAGCTCCCCCGAGCAGTCCTGGCCCAGCACTTTCAAGGCCACCTCCCCTCCCCATCTGGATGGGACTTCCAACCCTGAGAGCACCGTCCCTGAAAAAAAGATCCTCCTGCCCAACAACAATCATGTTACCAGTGACACAGGGGAAACAGAGGAGCGAGTTGTGGTGATCAGCAGCTCAGAGGATTCGGACACCGAGAATCTGTCCTCCCACGAGTTGGACGATAGCAGCAGTGAGTCCAGCAGCCTGCAGCTGGAGGGCCCCAATTCCCTCAAGGCCTTGGATGAGAGCCTCGCCGAACCCCACTTAGAAGACAGGACCCTGGTTTTCTTTGACCTCAAGATTGACAATGAAACCCAGAAAATTAGCCAGCTGGCCGCGGTGAACCGGGAAAGCAAGTTCCGTGTGCTCATCCAGCCCGAGGCCTTCAGTGTCTACTCCAAAGCTGTCTCCCTGGAGGCGGGGCTCCGGCACTTCCTCAGCTTCCTCACCACCATGCACCGTCCCATCTTGGCGTGTTCTAGGCTGTGGGGGCCGGGACTCCCCATCTTCTTCCAGACCCTGAGTGATATTAACAAGCTGTGGGAATTCCAGGACACCATCTCAGGTTTCTTGGCCGTGCTGCCCCTCATCCGGGAACGCATACCCGGCGCTAGCAGCTTCAAACTTGGGAACCTAGCCAAGACCTACCTGGCGAGAAACATGAGCGAACGCAGCGCTCTGGCTTCTGTGCTGGCCATGAGGGACTTGTGCTGCCTCCTTGAGATCTCCCCAGGACTGCCGCTGGCCCAGCATATCTACTCCTTTAGTAGCTTGCAGTGTTTTGCTTCCCTGCAGCCCCTAATTCAGGCCAGCGTCCTGCCACAGTCCGAGGCCCGCCTCTTGGCCCTCCACAATGTGAGCTTTGTAGAGTTACTGAATGCATATCGCACCAACAGGCAAGAAGGCTTGAAGAAGTATGTCCACTATCTGAGCCTGCAGACCACCCCGTTGTCATCGTCGGCTTCCACCCAAGTTGCCCAATTCCTGCAGGCTCTTAGCACCCACATGGAAGGACTGTTGGAAGGCCATGCCCCTGCTGGGGCAGAAGGCAAAGCTGAAAGTAAGGGATGCCTGGCCTAG

>NM_001311088.2:44-1942 Mus musculus promyelocytic leukemia (Pml), transcript variant 3, mRNA

ATGGAAACTGAACCAGTTTCCGTGCAGAAGGTACCTGCACCCCCTGGATCTCCCTGTCGACAACAGGACTCTGCCCTGACCCCCACGCCCACCATGCCTCCCCCAGAGGAACCCTCCGAAGACTATGAACACAGCCAAAGCCCTGCAGAGCAAGCCATACAGGAGGAATTTCAGTTTCTGCGCTGCCCGAGCTGCCAGGCCCAAGCCAAGTGCCCCAAACTGCTGCCTTGCCTGCACACGCTGTGCTCCGGATGCCTGGAGGCGCCTGGCCTGCAGTGCCCCATCTGCAAGGCTCCTGGGCAGGCCGATGCTAATGGGGAGGCCCTGGATAACGTGTTCTTCGAGAGCCTGCAGCGGCGCCTGGCGGTGTTCCGGCAGATCGTGGATGCTCAGGCTGCGTGCACCCGCTGCAAAGGCTTGGCCGACTTCTGGTGTTTCGAGTGTGAACAGCTCATTTGCAGCAAGTGCTTCGAAGCACACCAGTGGTACCTCAAGCATGAGGCCCGGCCCCTGGCTGATCTCCGCGACAATTCAGTGAGCAGCTTCCTCGACAGTACGCGCAAGTCCAATATCTTCTGCTCCAATACCAACCACCGCAACCCTGCGCTGACTGACATCTACTGCCGAGGCTGCGCCAAGCCTCTGTGTTGCACATGCGCGCTCCTGGACCGCAACCACAGCCATCTCCATTGCGATATTGGTGAGGAGATTCAGCAGTGGCATGAGGAACTAGGCACCATGACACAGACTCTGGAGGAGCAGGGCAGAACCTTCGACAGTGCCCATGCACAGATGTGCTCAGCTATAGGACAGCTGGACCACGCACGCGCAGACATTGAGAAGCAGATCCGCGCACGCGTGCGCCAGGTGGTAGACTACGTGCAGGCTCAGGAGCGCGAGCTGCTCGAGGCGGTGAATGACCGCTACCAGCGCGACTACCAGGAAATAGCTGGCCAGCTGAGCTGCCTGGAAGCTGTGCTGCAGCGCATCCGCACTAGTGGGGCGCTGGTCAAGAGGATGAAGCTCTATGCCTCCGACCAGGAGGTGCTGGATATGCACAGCTTTCTGCGCAAGGCACTCTGTAGCTTGCGCCAGGAGGAGCCCCAGAACCAGAAAGTCCAGCTGCTCACCAGAGGTTTCGAGGAGTTCAAGCTGTGCCTGCAGGACTTCATCTCCTGCATCACCCAGAGGATAAATGCAGCTGTAGCCAGCCCAGAGGCAGCCAGCAATCAACCAGAGGCAGCCAGCACTCACCCAGTGACAACCAGCACGCCTGAGGACCTTGAGCAGCCCAAAGAAGTGCAGAGTGTACAGGCTCAGGCCCTAGAGCTGTCTAAGACCCAACCTGTGGCTATGGTAAAAACAGTGCCTGGAGCACACCCTGTACCAGTGTATGCCTTTTCAATGCAAGGCCCCACCTACAGAGAGGAGGCTTCTCAGACAGTCGGCTCCATGAAGAGGAAGTGCTCCCACGAAGATTGCTCCAGGAAGATCATCAAGATGGAGTCCACAGAGGAGAACGAGGACAGGTTGGCCACAAGCTCCCCCGAGCAGTCCTGGCCCAGCACTTTCAAGGCCACCTCCCCTCCCCATCTGGATGGGACTTCCAACCCTGAGAGCACCGTCCCTGAAAAAAAGATCCTCCTGCCCAACAACAATCATGTTACCAGTGACACAGGGGAAACAGAGGAGCGAGTTGTGGTGATCAGCAGCTCAGAGGATTCGGACACCGAGAATCTGTCCTCCCACGAGTTGGACGATAGCAGCAGTGAGTCCAGCAGCCTGCAGCTGGAGGGCCCCAATTCCCTCAAGGCCTTGGATGAGAGCCTCGCCGAACCCCACTTAGAAGACAGGACCCTGGTTTTCTTTGACCTCAAGATTGACAATGAAAAAAATTAG

>XM_006510860.3:105-2936 PREDICTED: Mus musculus promyelocytic leukemia (Pml), transcript variant X1, mRNA

ATGGAAACTGAACCAGTTTCCGTGCAGAAGGTACCTGCACCCCCTGGATCTCCCTGTCGACAACAGGACTCTGCCCTGACCCCCACGCCCACCATGCCTCCCCCAGAGGAACCCTCCGAAGACTATGAACACAGCCAAAGCCCTGCAGAGCAAGCCATACAGGAGGAATTTCAGTTTCTGCGCTGCCCGAGCTGCCAGGCCCAAGCCAAGTGCCCCAAACTGCTGCCTTGCCTGCACACGCTGTGCTCCGGATGCCTGGAGGCGCCTGGCCTGCAGTGCCCCATCTGCAAGGCTCCTGGGCAGGCCGATGCTAATGGGGAGGCCCTGGATAACGTGTTCTTCGAGAGCCTGCAGCGGCGCCTGGCGGTGTTCCGGCAGATCGTGGATGCTCAGGCTGCGTGCACCCGCTGCAAAGGCTTGGCCGACTTCTGGTGTTTCGAGTGTGAACAGCTCATTTGCAGCAAGTGCTTCGAAGCACACCAGTGGTACCTCAAGCATGAGGCCCGGCCCCTGGCTGATCTCCGCGACAATTCAGTGAGCAGCTTCCTCGACAGTACGCGCAAGTCCAATATCTTCTGCTCCAATACCAACCACCGCAACCCTGCGCTGACTGACATCTACTGCCGAGGCTGCGCCAAGCCTCTGTGTTGCACATGCGCGCTCCTGGACCGCAACCACAGCCATCTCCATTGCGATATTGGTGAGGAGATTCAGCAGTGGCATGAGGAACTAGGCACCATGACACAGACTCTGGAGGAGCAGGGCAGAACCTTCGACAGTGCCCATGCACAGATGTGCTCAGCTATAGGACAGCTGGACCACGCACGCGCAGACATTGAGAAGCAGATCCGCGCACGCGTGCGCCAGGTGGTAGACTACGTGCAGGCTCAGGAGCGCGAGCTGCTCGAGGCGGTGAATGACCGCTACCAGCGCGACTACCAGGAAATAGCTGGCCAGCTGAGCTGCCTGGAAGCTGTGCTGCAGCGCATCCGCACTAGTGGGGCGCTGGTCAAGAGGATGAAGCTCTATGCCTCCGACCAGGAGGTGCTGGATATGCACAGCTTTCTGCGCAAGGCACTCTGTAGCTTGCGCCAGGAGGAGCCCCAGAACCAGAAAGTCCAGCTGCTCACCAGAGGTTTCGAGGAGTTCAAGCTGTGCCTGCAGGACTTCATCTCCTGCATCACCCAGAGGATAAATGCAGCTGTAGCCAGCCCAGAGGCAGCCAGCAATCAACCAGAGGCAGCCAGCACTCACCCAGTGACAACCAGCACGCCTGAGGACCTTGAGCAGCCCAAAGAAGTGCAGAGTGTACAGGCTCAGGCCCTAGAGCTGTCTAAGACCCAACCTGTGGCTATGGTAAAAACAGTGCCTGGAGCACACCCTGTACCAGTGTATGCCTTTTCAATGCAAGGCCCCACCTACAGAGAGGAGGCTTCTCAGACAGTCGGCTCCATGAAGAGGAAGTGCTCCCACGAAGATTGCTCCAGGAAGATCATCAAGATGGAGTCCACAGAGGAGAACGAGGACAGGTTGGCCACAAGCTCCCCCGAGCAGTCCTGGCCCAGCACTTTCAAGGCCACCTCCCCTCCCCATCTGGATGGGACTTCCAACCCTGAGAGCACCGTCCCTGAAAAAAAGATCCTCCTGCCCAACAACAATCATGTTACCAGTGACACAGGGGAAACAGAGGAGCGAGTTGTGGTGATCAGCAGCTCAGAGGATTCGGACACCGAGAATCTGTCCTCCCACGAGTTGGACGATAGCAGCAGTGAGTCCAGCAGCCTGCAGCTGGAGGGCCCCAATTCCCTCAAGGCCTTGGATGAGAGCCTCGCCGAACCCCACTTAGAAGACAGGACCCTGGTTTTCTTTGACCTCAAGATTGACAATGAAACCCAGAAAATTAGCCAGCTGGCCGCGGTGAACCGGGAAAGCAAGTTCCGTGTGCTCATCCAGCCCGAGGCCTTCAGTGTCTACTCCAAAGCTGTCTCCCTGGAGGCGGGGCTCCGGCACTTCCTCAGCTTCCTCACCACCATGCACCGTCCCATCTTGGCGTGTTCTAGGCTGTGGGGGCCGGGACTCCCCATCTTCTTCCAGACCCTGAGTGATATTAACAAGCTGTGGGAATTCCAGGACACCATCTCAGGTTTCTTGGCCGTGCTGCCCCTCATCCGGGAACGCATACCCGGCGCTAGCAGCTTCAAACTTGGGAACCTAGCCAAGACCTACCTGGCGAGAAACATGAGCGAACGCAGCGCTCTGGCTTCTGTGCTGGCCATGAGGGACTTGTGCTGCCTCCTTGAGATCTCCCCAGGACTGCCGCTGGCCCAGCATATCTACTCCTTTAGTAGCTTGCAGTGTTTTGCTTCCCTGCAGCCCCTAATTCAGGCCAGCGTCCTGCCACAGTCCGAGGCCCGCCTCTTGGCCCTCCACAATGTGAGCTTTGTAGAGTTACTGAATGCATATCGCACCAACAGGCAAGAAGGCTTGAAGAAGTATGTCCACTATCTGAGCCTGCAGACCACCCCGTTGTCATCGTCGGCTTCCACCCAAGTTGCCCAATTCCTGCAGGCTCTTAGCACCCACATGGAAGGACTGTTGGAAGGCCATGCCCCTGCTGGGGCAGAAGGCAAAGCTGAAACAGACACCAGAGGCACCCCAGACCTAGACCTCCCTTCTAAAAGCCAGAACCAGGAAGGCTTGCATAAAGAAGGTGTGGCCTCTGAGCTCTCAAGGGCCCCAGTTGCCACCAACCACCACCACAGGTGGCCTTATGGCTCTTCCTGGATGTTCGATTCATGGTGTTTTCCAGATAGCCCAGAGTTCCAGCTTTGA

>XM_006510861.3:105-2798 PREDICTED: Mus musculus promyelocytic leukemia (Pml), transcript variant X2, mRNA

ATGGAAACTGAACCAGTTTCCGTGCAGAAGGTACCTGCACCCCCTGGATCTCCCTGTCGACAACAGGACTCTGCCCTGACCCCCACGCCCACCATGCCTCCCCCAGAGGAACCCTCCGAAGACTATGAACACAGCCAAAGCCCTGCAGAGCAAGCCATACAGGAGGAATTTCAGTTTCTGCGCTGCCCGAGCTGCCAGGCCCAAGCCAAGTGCCCCAAACTGCTGCCTTGCCTGCACACGCTGTGCTCCGGATGCCTGGAGGCGCCTGGCCTGCAGTGCCCCATCTGCAAGGCTCCTGGGCAGGCCGATGCTAATGGGGAGGCCCTGGATAACGTGTTCTTCGAGAGCCTGCAGCGGCGCCTGGCGGTGTTCCGGCAGATCGTGGATGCTCAGGCTGCGTGCACCCGCTGCAAAGGCTTGGCCGACTTCTGGTGTTTCGAGTGTGAACAGCTCATTTGCAGCAAGTGCTTCGAAGCACACCAGTGGTACCTCAAGCATGAGGCCCGGCCCCTGGCTGATCTCCGCGACAATTCAGTGAGCAGCTTCCTCGACAGTACGCGCAAGTCCAATATCTTCTGCTCCAATACCAACCACCGCAACCCTGCGCTGACTGACATCTACTGCCGAGGCTGCGCCAAGCCTCTGTGTTGCACATGCGCGCTCCTGGACCGCAACCACAGCCATCTCCATTGCGATATTGGTGAGGAGATTCAGCAGTGGCATGAGGAACTAGGCACCATGACACAGACTCTGGAGGAGCAGGGCAGAACCTTCGACAGTGCCCATGCACAGATGTGCTCAGCTATAGGACAGCTGGACCACGCACGCGCAGACATTGAGAAGCAGATCCGCGCACGCGTGCGCCAGGTGGTAGACTACGTGCAGGCTCAGGAGCGCGAGCTGCTCGAGGCGGTGAATGACCGCTACCAGCGCGACTACCAGGAAATAGCTGGCCAGCTGAGCTGCCTGGAAGCTGTGCTGCAGCGCATCCGCACTAGTGGGGCGCTGGTCAAGAGGATGAAGCTCTATGCCTCCGACCAGGAGGTGCTGGATATGCACAGCTTTCTGCGCAAGGCACTCTGTAGCTTGCGCCAGGAGGAGCCCCAGAACCAGAAAGTCCAGCTGCTCACCAGAGGTTTCGAGGAGTTCAAGCTGTGCCTGCAGGACTTCATCTCCTGCATCACCCAGAGGATAAATGCAGCTGTAGCCAGCCCAGAGGCAGCCAGCAATCAACCAGAGGCAGCCAGCACTCACCCAGTGACAACCAGCACGCCTGAGGACCTTGAGCAGGAGGCTTCTCAGACAGTCGGCTCCATGAAGAGGAAGTGCTCCCACGAAGATTGCTCCAGGAAGATCATCAAGATGGAGTCCACAGAGGAGAACGAGGACAGGTTGGCCACAAGCTCCCCCGAGCAGTCCTGGCCCAGCACTTTCAAGGCCACCTCCCCTCCCCATCTGGATGGGACTTCCAACCCTGAGAGCACCGTCCCTGAAAAAAAGATCCTCCTGCCCAACAACAATCATGTTACCAGTGACACAGGGGAAACAGAGGAGCGAGTTGTGGTGATCAGCAGCTCAGAGGATTCGGACACCGAGAATCTGTCCTCCCACGAGTTGGACGATAGCAGCAGTGAGTCCAGCAGCCTGCAGCTGGAGGGCCCCAATTCCCTCAAGGCCTTGGATGAGAGCCTCGCCGAACCCCACTTAGAAGACAGGACCCTGGTTTTCTTTGACCTCAAGATTGACAATGAAACCCAGAAAATTAGCCAGCTGGCCGCGGTGAACCGGGAAAGCAAGTTCCGTGTGCTCATCCAGCCCGAGGCCTTCAGTGTCTACTCCAAAGCTGTCTCCCTGGAGGCGGGGCTCCGGCACTTCCTCAGCTTCCTCACCACCATGCACCGTCCCATCTTGGCGTGTTCTAGGCTGTGGGGGCCGGGACTCCCCATCTTCTTCCAGACCCTGAGTGATATTAACAAGCTGTGGGAATTCCAGGACACCATCTCAGGTTTCTTGGCCGTGCTGCCCCTCATCCGGGAACGCATACCCGGCGCTAGCAGCTTCAAACTTGGGAACCTAGCCAAGACCTACCTGGCGAGAAACATGAGCGAACGCAGCGCTCTGGCTTCTGTGCTGGCCATGAGGGACTTGTGCTGCCTCCTTGAGATCTCCCCAGGACTGCCGCTGGCCCAGCATATCTACTCCTTTAGTAGCTTGCAGTGTTTTGCTTCCCTGCAGCCCCTAATTCAGGCCAGCGTCCTGCCACAGTCCGAGGCCCGCCTCTTGGCCCTCCACAATGTGAGCTTTGTAGAGTTACTGAATGCATATCGCACCAACAGGCAAGAAGGCTTGAAGAAGTATGTCCACTATCTGAGCCTGCAGACCACCCCGTTGTCATCGTCGGCTTCCACCCAAGTTGCCCAATTCCTGCAGGCTCTTAGCACCCACATGGAAGGACTGTTGGAAGGCCATGCCCCTGCTGGGGCAGAAGGCAAAGCTGAAACAGACACCAGAGGCACCCCAGACCTAGACCTCCCTTCTAAAAGCCAGAACCAGGAAGGCTTGCATAAAGAAGGTGTGGCCTCTGAGCTCTCAAGGGCCCCAGTTGCCACCAACCACCACCACAGGTGGCCTTATGGCTCTTCCTGGATGTTCGATTCATGGTGTTTTCCAGATAGCCCAGAGTTCCAGCTTTGA

>XM_006510862.5:105-2042 PREDICTED: Mus musculus promyelocytic leukemia (Pml), transcript variant X3, mRNA

ATGGAAACTGAACCAGTTTCCGTGCAGAAGGTACCTGCACCCCCTGGATCTCCCTGTCGACAACAGGACTCTGCCCTGACCCCCACGCCCACCATGCCTCCCCCAGAGGAACCCTCCGAAGACTATGAACACAGCCAAAGCCCTGCAGAGCAAGCCATACAGGAGGAATTTCAGTTTCTGCGCTGCCCGAGCTGCCAGGCCCAAGCCAAGTGCCCCAAACTGCTGCCTTGCCTGCACACGCTGTGCTCCGGATGCCTGGAGGCGCCTGGCCTGCAGTGCCCCATCTGCAAGGCTCCTGGGCAGGCCGATGCTAATGGGGAGGCCCTGGATAACGTGTTCTTCGAGAGCCTGCAGCGGCGCCTGGCGGTGTTCCGGCAGATCGTGGATGCTCAGGCTGCGTGCACCCGCTGCAAAGGCTTGGCCGACTTCTGGTGTTTCGAGTGTGAACAGCTCATTTGCAGCAAGTGCTTCGAAGCACACCAGTGGTACCTCAAGCATGAGGCCCGGCCCCTGGCTGATCTCCGCGACAATTCAGTGAGCAGCTTCCTCGACAGTACGCGCAAGTCCAATATCTTCTGCTCCAATACCAACCACCGCAACCCTGCGCTGACTGACATCTACTGCCGAGGCTGCGCCAAGCCTCTGTGTTGCACATGCGCGCTCCTGGACCGCAACCACAGCCATCTCCATTGCGATATTGGTGAGGAGATTCAGCAGTGGCATGAGGAACTAGGCACCATGACACAGACTCTGGAGGAGCAGGGCAGAACCTTCGACAGTGCCCATGCACAGATGTGCTCAGCTATAGGACAGCTGGACCACGCACGCGCAGACATTGAGAAGCAGATCCGCGCACGCGTGCGCCAGGTGGTAGACTACGTGCAGGCTCAGGAGCGCGAGCTGCTCGAGGCGGTGAATGACCGCTACCAGCGCGACTACCAGGAAATAGCTGGCCAGCTGAGCTGCCTGGAAGCTGTGCTGCAGCGCATCCGCACTAGTGGGGCGCTGGTCAAGAGGATGAAGCTCTATGCCTCCGACCAGGAGGTGCTGGATATGCACAGCTTTCTGCGCAAGGCACTCTGTAGCTTGCGCCAGGAGGAGCCCCAGAACCAGAAAGTCCAGCTGCTCACCAGAGGTTTCGAGGAGTTCAAGCTGTGCCTGCAGGACTTCATCTCCTGCATCACCCAGAGGATAAATGCAGCTGTAGCCAGCCCAGAGGCAGCCAGCAATCAACCAGAGGCAGCCAGCACTCACCCAGTGACAACCAGCACGCCTGAGGACCTTGAGCAGCCCAAAGAAGTGCAGAGTGTACAGGCTCAGGCCCTAGAGCTGTCTAAGACCCAACCTGTGGCTATGGTAAAAACAGTGCCTGGAGCACACCCTGTACCAGTGTATGCCTTTTCAATGCAAGGCCCCACCTACAGAGAGGAGGCTTCTCAGACAGTCGGCTCCATGAAGAGGAAGTGCTCCCACGAAGATTGCTCCAGGAAGATCATCAAGATGGAGTCCACAGAGGAGAACGAGGACAGGTTGGCCACAAGCTCCCCCGAGCAGTCCTGGCCCAGCACTTTCAAGGCCACCTCCCCTCCCCATCTGGATGGGACTTCCAACCCTGAGAGCACCGTCCCTGAAAAAAAGATCCTCCTGCCCAACAACAATCATGTTACCAGTGACACAGGGGAAACAGAGGAGCGAGTTGTGGTGATCAGCAGCTCAGAGGATTCGGACACCGAGAATCTGGGACACTTCTCTGAGCTTCATCCTGACCCTGACCCTAGCCTGTTGACTATACCTTCTGCTCCTGGTAAGAAACTGAAGCCCTTTTGGCGGTGTGGGAATTGTCAGCATCTGCTACCTAATTACAGATTTGTCTGCTGTTGGCACTTCCCTTCATCTGCCACTTCTGCCTGGTTGGAGGGGAAGTCTGTCTTCCTCTAA

>XM_006510864.5:104-1873 PREDICTED: Mus musculus promyelocytic leukemia (Pml), transcript variant X4, mRNA

ATGGAAACTGAACCAGTTTCCGTGCAGAAGGTACCTGCACCCCCTGGATCTCCCTGTCGACAACAGGACTCTGCCCTGACCCCCACGCCCACCATGCCTCCCCCAGAGGAACCCTCCGAAGACTATGAACACAGCCAAAGCCCTGCAGAGCAAGCCATACAGGAGGAATTTCAGTTTCTGCGCTGCCCGAGCTGCCAGGCCCAAGCCAAGTGCCCCAAACTGCTGCCTTGCCTGCACACGCTGTGCTCCGGATGCCTGGAGGCGCCTGGCCTGCAGTGCCCCATCTGCAAGGCTCCTGGGCAGGCCGATGCTAATGGGGAGGCCCTGGATAACGTGTTCTTCGAGAGCCTGCAGCGGCGCCTGGCGGTGTTCCGGCAGATCGTGGATGCTCAGGCTGCGTGCACCCGCTGCAAAGGCTTGGCCGACTTCTGGTGTTTCGAGTGTGAACAGCTCATTTGCAGCAAGTGCTTCGAAGCACACCAGTGGTACCTCAAGCATGAGGCCCGGCCCCTGGCTGATCTCCGCGACAATTCAGTGAGCAGCTTCCTCGACAGTACGCGCAAGTCCAATATCTTCTGCTCCAATACCAACCACCGCAACCCTGCGCTGACTGACATCTACTGCCGAGGCTGCGCCAAGCCTCTGTGTTGCACATGCGCGCTCCTGGACCGCAACCACAGCCATCTCCATTGCGATATTGGTGAGGAGATTCAGCAGTGGCATGAGGAACTAGGCACCATGACACAGACTCTGGAGGAGCAGGGCAGAACCTTCGACAGTGCCCATGCACAGATGTGCTCAGCTATAGGACAGCTGGACCACGCACGCGCAGACATTGAGAAGCAGATCCGCGCACGCGTGCGCCAGGTGGTAGACTACGTGCAGGCTCAGGAGCGCGAGCTGCTCGAGGCGGTGAATGACCGCTACCAGCGCGACTACCAGGAAATAGCTGGCCAGCTGAGCTGCCTGGAAGCTGTGCTGCAGCGCATCCGCACTAGTGGGGCGCTGGTCAAGAGGATGAAGCTCTATGCCTCCGACCAGGAGGTGCTGGATATGCACAGCTTTCTGCGCAAGGCACTCTGTAGCTTGCGCCAGGAGGAGCCCCAGAACCAGAAAGTCCAGCTGCTCACCAGAGGTTTCGAGGAGTTCAAGCTGTGCCTGCAGGACTTCATCTCCTGCATCACCCAGAGGATAAATGCAGCTGTAGCCAGCCCAGAGGCAGCCAGCAATCAACCAGAGGCAGCCAGCACTCACCCAGTGACAACCAGCACGCCTGAGGACCTTGAGCAGCCCAAAGAAGTGCAGAGTGTACAGGCTCAGGCCCTAGAGCTGTCTAAGACCCAACCTGTGGCTATGGTAAAAACAGTGCCTGGAGCACACCCTGTACCAGTGTATGCCTTTTCAATGCAAGGCCCCACCTACAGAGAGGAGGCTTCTCAGACAGTCGGCTCCATGAAGAGGAAGTGCTCCCACGAAGATTGCTCCAGGAAGATCATCAAGATGGAGTCCACAGAGGAGAACGAGGACAGGTTGGCCACAAGCTCCCCCGAGCAGTCCTGGCCCAGCACTTTCAAGGCCACCTCCCCTCCCCATCTGGATGGGACTTCCAACCCTGAGAGCACCGTCCCTGAAAAAAAGATCCTCCTGCCCAACAACAATCATGTTACCAGTGACACAGGGGAAACAGAGGAGCGAGTTGTGGTGATCAGCAGCTCAGAGGATTCGGACACCGAGAATCTGCCCAGAAAATTAGCCAGCTGGCCGCGGTGA

>XM_030244128.2:104-1864 PREDICTED: Mus musculus promyelocytic leukemia (Pml), transcript variant X5, mRNA

ATGGAAACTGAACCAGTTTCCGTGCAGAAGGTACCTGCACCCCCTGGATCTCCCTGTCGACAACAGGACTCTGCCCTGACCCCCACGCCCACCATGCCTCCCCCAGAGGAACCCTCCGAAGACTATGAACACAGCCAAAGCCCTGCAGAGCAAGCCATACAGGAGGAATTTCAGTTTCTGCGCTGCCCGAGCTGCCAGGCCCAAGCCAAGTGCCCCAAACTGCTGCCTTGCCTGCACACGCTGTGCTCCGGATGCCTGGAGGCGCCTGGCCTGCAGTGCCCCATCTGCAAGGCTCCTGGGCAGGCCGATGCTAATGGGGAGGCCCTGGATAACGTGTTCTTCGAGAGCCTGCAGCGGCGCCTGGCGGTGTTCCGGCAGATCGTGGATGCTCAGGCTGCGTGCACCCGCTGCAAAGGCTTGGCCGACTTCTGGTGTTTCGAGTGTGAACAGCTCATTTGCAGCAAGTGCTTCGAAGCACACCAGTGGTACCTCAAGCATGAGGCCCGGCCCCTGGCTGATCTCCGCGACAATTCAGTGAGCAGCTTCCTCGACAGTACGCGCAAGTCCAATATCTTCTGCTCCAATACCAACCACCGCAACCCTGCGCTGACTGACATCTACTGCCGAGGCTGCGCCAAGCCTCTGTGTTGCACATGCGCGCTCCTGGACCGCAACCACAGCCATCTCCATTGCGATATTGGTGAGGAGATTCAGCAGTGGCATGAGGAACTAGGCACCATGACACAGACTCTGGAGGAGCAGGGCAGAACCTTCGACAGTGCCCATGCACAGATGTGCTCAGCTATAGGACAGCTGGACCACGCACGCGCAGACATTGAGAAGCAGATCCGCGCACGCGTGCGCCAGGTGGTAGACTACGTGCAGGCTCAGGAGCGCGAGCTGCTCGAGGCGGTGAATGACCGCTACCAGCGCGACTACCAGGAAATAGCTGGCCAGCTGAGCTGCCTGGAAGCTGTGCTGCAGCGCATCCGCACTAGTGGGGCGCTGGTCAAGAGGATGAAGCTCTATGCCTCCGACCAGGAGGTGCTGGATATGCACAGCTTTCTGCGCAAGGCACTCTGTAGCTTGCGCCAGGAGGAGCCCCAGAACCAGAAAGTCCAGCTGCTCACCAGAGGTTTCGAGGAGTTCAAGCTGTGCCTGCAGGACTTCATCTCCTGCATCACCCAGAGGATAAATGCAGCTGTAGCCAGCCCAGAGGCAGCCAGCAATCAACCAGAGGCAGCCAGCACTCACCCAGTGACAACCAGCACGCCTGAGGACCTTGAGCAGGAGGCTTCTCAGACAGTCGGCTCCATGAAGAGGAAGTGCTCCCACGAAGATTGCTCCAGGAAGATCATCAAGATGGAGTCCACAGAGGAGAACGAGGACAGGTTGGCCACAAGCTCCCCCGAGCAGTCCTGGCCCAGCACTTTCAAGGCCACCTCCCCTCCCCATCTGGATGGGACTTCCAACCCTGAGAGCACCGTCCCTGAAAAAAAGATCCTCCTGCCCAACAACAATCATGTTACCAGTGACACAGGGGAAACAGAGGAGCGAGTTGTGGTGATCAGCAGCTCAGAGGATTCGGACACCGAGAATCTGTCCTCCCACGAGTTGGACGATAGCAGCAGTGAGTCCAGCAGCCTGCAGCTGGAGGGCCCCAATTCCCTCAAGGCCTTGGATGAGAGCCTCGCCGAACCCCACTTAGAAGACAGGACCCTGGTTTTCTTTGACCTCAAGATTGACAATGAAAAAAATTAG

>XM_017313212.3:104-1735 PREDICTED: Mus musculus promyelocytic leukemia (Pml), transcript variant X6, mRNA

ATGGAAACTGAACCAGTTTCCGTGCAGAAGGTACCTGCACCCCCTGGATCTCCCTGTCGACAACAGGACTCTGCCCTGACCCCCACGCCCACCATGCCTCCCCCAGAGGAACCCTCCGAAGACTATGAACACAGCCAAAGCCCTGCAGAGCAAGCCATACAGGAGGAATTTCAGTTTCTGCGCTGCCCGAGCTGCCAGGCCCAAGCCAAGTGCCCCAAACTGCTGCCTTGCCTGCACACGCTGTGCTCCGGATGCCTGGAGGCGCCTGGCCTGCAGTGCCCCATCTGCAAGGCTCCTGGGCAGGCCGATGCTAATGGGGAGGCCCTGGATAACGTGTTCTTCGAGAGCCTGCAGCGGCGCCTGGCGGTGTTCCGGCAGATCGTGGATGCTCAGGCTGCGTGCACCCGCTGCAAAGGCTTGGCCGACTTCTGGTGTTTCGAGTGTGAACAGCTCATTTGCAGCAAGTGCTTCGAAGCACACCAGTGGTACCTCAAGCATGAGGCCCGGCCCCTGGCTGATCTCCGCGACAATTCAGTGAGCAGCTTCCTCGACAGTACGCGCAAGTCCAATATCTTCTGCTCCAATACCAACCACCGCAACCCTGCGCTGACTGACATCTACTGCCGAGGCTGCGCCAAGCCTCTGTGTTGCACATGCGCGCTCCTGGACCGCAACCACAGCCATCTCCATTGCGATATTGGTGAGGAGATTCAGCAGTGGCATGAGGAACTAGGCACCATGACACAGACTCTGGAGGAGCAGGGCAGAACCTTCGACAGTGCCCATGCACAGATGTGCTCAGCTATAGGACAGCTGGACCACGCACGCGCAGACATTGAGAAGCAGATCCGCGCACGCGTGCGCCAGGTGGTAGACTACGTGCAGGCTCAGGAGCGCGAGCTGCTCGAGGCGGTGAATGACCGCTACCAGCGCGACTACCAGGAAATAGCTGGCCAGCTGAGCTGCCTGGAAGCTGTGCTGCAGCGCATCCGCACTAGTGGGGCGCTGGTCAAGAGGATGAAGCTCTATGCCTCCGACCAGGAGGTGCTGGATATGCACAGCTTTCTGCGCAAGGCACTCTGTAGCTTGCGCCAGGAGGAGCCCCAGAACCAGAAAGTCCAGCTGCTCACCAGAGGTTTCGAGGAGTTCAAGCTGTGCCTGCAGGACTTCATCTCCTGCATCACCCAGAGGATAAATGCAGCTGTAGCCAGCCCAGAGGCAGCCAGCAATCAACCAGAGGCAGCCAGCACTCACCCAGTGACAACCAGCACGCCTGAGGACCTTGAGCAGGAGGCTTCTCAGACAGTCGGCTCCATGAAGAGGAAGTGCTCCCACGAAGATTGCTCCAGGAAGATCATCAAGATGGAGTCCACAGAGGAGAACGAGGACAGGTTGGCCACAAGCTCCCCCGAGCAGTCCTGGCCCAGCACTTTCAAGGCCACCTCCCCTCCCCATCTGGATGGGACTTCCAACCCTGAGAGCACCGTCCCTGAAAAAAAGATCCTCCTGCCCAACAACAATCATGTTACCAGTGACACAGGGGAAACAGAGGAGCGAGTTGTGGTGATCAGCAGCTCAGAGGATTCGGACACCGAGAATCTGCCCAGAAAATTAGCCAGCTGGCCGCGGTGA

>Seq-mPmlX~~K~~7[organism=Mus musculus][cell-line=MEF][moltype=mRNA]

ATGGAAACTGAACCAGTTTCCGTGCAGAAGGTACCTGCACCCCCTGGATCTCCCTGTCGACAACAGGACTCTGCCCTGACCCCCACGCCCACCATGCCTCCCCCAGAGGAACCCTCCGAAGACTATGAACACAGCCAAAGCCCTGCAGAGCAAGCCATACAGGAGGAATTTCAGTTTCTGCGCTGCCCGAGCTGCCAGGCCCAAGCCAAGTGCCCCAAACTGCTGCCTTGCCTGCACACGCTGTGCTCCGGATGCCTGGAGGCGCCTGGCCTGCAGTGCCCCATCTGCAAGGCTCCTGGGCAGGCCGATGCTAATGGGGAGGCCCTGGATAACGTGTTCTTCGAGAGCCTGCAGCGGCGCCTGGCGGTGTTCCGGCAGATCGTGGATGCTCAGGCTGCGTGCACCCGCTGCAAAGGCTTGGCCGACTTCTGGTGTTTCGAGTGTGAACAGCTCATTTGCAGCAAGTGCTTCGAAGCACACCAGTGGTACCTCAAGCATGAGGCCCGGCCCCTGGCTGATCTCCGCGACAATTCAGTGAGCAGCTTCCTCGACAGTACGCGCAAGTCCAATATCTTCTGCTCCAATACCAACCACCGCAACCCTGCGCTGACTGACATCTACTGCCGAGGCTGCGCCAAGCCTCTGTGTTGCACATGCGCGCTCCTGGACCGCAACCACAGCCATCTCCATTGCGATATTGATGCAGCTGTAGCCAGCCCAGAGGCAGCCAGCAATCAACCAGAGGCAGCCAGCACTCACCCAGTGACAACCAGCACGCCTGAGGACCTTGAGCAGGAGGCTTCTCAGACAGTCGGCTCCATGAAGAGGAAGTGCTCCCACGAAGATTGCTCCAGGAAGATCATCAAGATGGAGTCCACAGAGGAGAACGAGGACAGGTTGGCCACAAGCTCCCCCGAGCAGTCCTGGCCCAGCACTTTCAAGGCCACCTCCCCTCCCCATCTGGATGGGACTTCCAACCCTGAGAGCACCGTCCCTGAAAAAAAGATCCTCCTGCCCAACAACAATCATGTTACCAGTGACACAGGGGAAACAGAGGAGCGAGTTGTGGTGATCAGCAGCTCAGAGGATTCGGACACCGAGAATCTGTCCTCCCACGAGTTGGACGATAGCAGCAGTGAGTCCAGCAGCCTGCAGCTGGAGGGCCCCAATTCCCTCAAGGCCTTGGATGAGAGCCTCGCCGAACCCCACTTAGAAGACAGGACCCTGGTTTTCTTTGACCTCAAGATTGACAATGAAACCCAGAAAATTAGCCAGCTGGCCGCGGTGAACCGGGAAAGCAAGTTCCGTGTGCTCATCCAGCCCGAGGCCTTCAGTGTCTACTCCAAAGCTGTCTCCCTGGAGGCGGGGCTCCGGCACTTCCTCAGCTTCCTCACCACCATGCACCGTCCCATCTTGGCGTGTTCTAGGCTGTGGGGGCCGGGACTCCCCATCTTCTTCCAGACCCTGAGTGATATTAACAAGCTGTGGGAATTCCAGGACACCATCTCAGGTTTCTTGGCCGTGCTGCCCCTCATCCGGGAACGCATACCCGGCGCTAGCAGCTTCAAACTTGGGAACCTAGCCAAGACCTACCTGGCGAGAAACATGAGCGAACGCAGCGCTCTGGCTTCTGTGCTGGCCATGAGGGACTTGTGCTGCCTCCTTGAGATCTCCCCAGGACTGCCGCTGGCCCAGCATATCTACTCCTTTAGTAGCTTGCAGTGTTTTGCTTCCCTGCAGCCCCTAATTCAGGCCAGCGTCCTGCCACAGTCCGAGGCCCGCCTCTTGGCCCTCCACAATGTGAGCTTTGTAGAGTTACTGAATGCATATCGCACCAACAGGCAAGAAGGCTTGAAGAAGTATGTCCACTATCTGAGCCTGCAGACCACCCCGTTGTCATCGTCGGCTTCCACCCAAGTTGCCCAATTCCTGCAGGCTCTTAGCACCCACATGGAAGGACTGTTGGAAGGCCATGCCCCTGCTGGGGCAGAAGGCAAAGCTGAAAGTAAGGGATGCCTGGCCTAG

**Figure S2.** Coding sequences of the three experimentally confirmed (mPML1 to mPML3) and six computationally predicted (mPMLX1 to mPMLX6) isoforms along the novel isoform mPMLX~~K~~7. Nucleotides missing in the Sanger-sequencing coverage of the mPMLX3 are underlined in the respective sequence).

**Figure S2 alt text:** Coding sequences of individual murine PML isoforms, including their NCBI accession numbers, coding ranges, and confirmed or predicted status. For the PMLX3 isoform, an underlined sequence indicates a segment missing in the respective Sanger sequencing data.

**
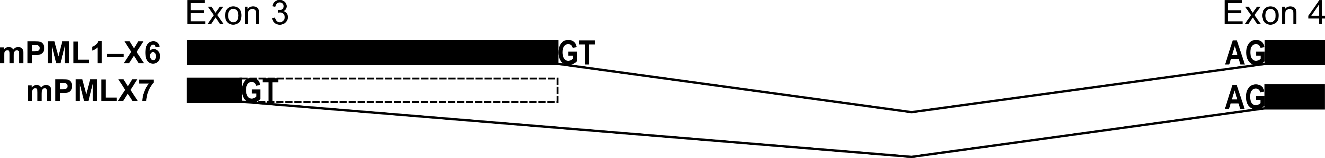
**

**Figure S3.** Schematic representation of the consensus splice site sequence within exon 3 of the novel mPMLX~~K~~7 isoform.

**Figure S3 alt text:** Schematic diagram displaying the novel donor splice site (GT) in murine PMLX7 isoform, producing a shortened exon 3 (solid box), compared with the canonical donor splice site in isoforms PML1 to PML X6 that generates a full-length exon 3 (dashed box). Splicing to the canonical acceptor splice site of exon 4 is illustrated by a chevron-shaped intron.

**
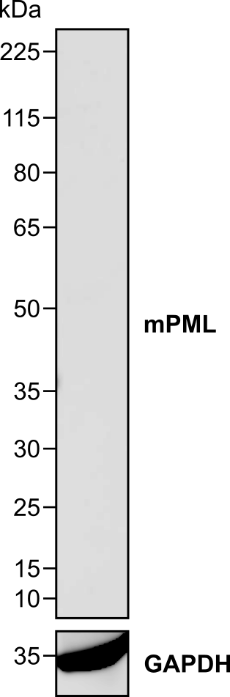
**

**Figure S4.** Validation of anti-PML antibody specificity in *Pml*^−/−^ MEFs. Whole-cell lysates were analyzed by western blotting using the anti-PML antibody with GAPDH as a loading control.

**Figure S4 alt text:** Western blot validating the specificity of the anti-PML antibody, showing no specific protein bands in PML-knockout control cells across the full molecular weight marker range.


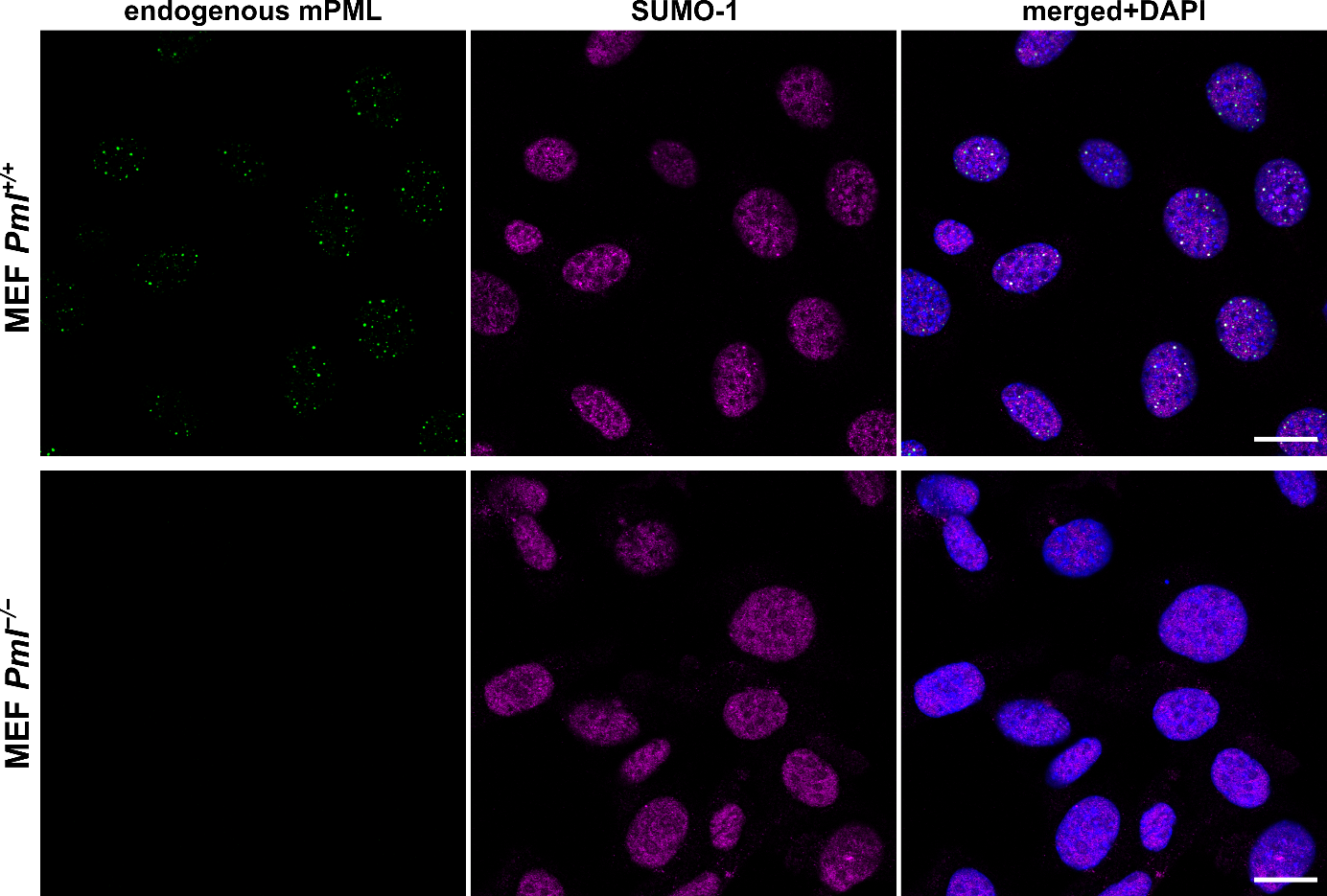


**Figure S5:** Endogenous mPML NBs formation and validation of *Pml*^−/−^ MEFs. Endogenous mPML proteins (green) and SUMO-1 proteins (magenta) were immunostained using specific antibod~~y~~ies. Cell nuclei were visualized by DAPI staining. Scale bar, 20 μm.

**Figure S5 alt text:** Confocal microscopy images, including single-channel (n=2) and merged views, showing dot-like endogenous murine PML nuclear bodies in PML wild-type cells and confirming the absence of PML protein in PML knockout cells.

**Table S3.** Cloning of mPML-tRFP/mPML-S expression constructs. The mPml3-tRFP plasmid was purchased from Eurofins Genomics Blue Heron as mPml3 inserted into a pCMV6-AC-RFP vector (PS100010) (OriGene). mPml1 and mPmlX~~K~~7 sequences were obtained by performing RT-PCR on RNA template from MEF cells and cloned into the pENTR/D-TOPO vector using the pENTR Directional TOPO Cloning Kit (Invitrogen). mPml1 was further cloned into the pCMV6-AC-RFP vector using NEBuilder HiFi DNA Assembly Cloning Kit (NEB). mPml2 was purchased as mPml2-tGFP plasmid (MG226141, OriGene) and cloned into the pCMV6-AC-RFP vector using *NotI* and *NdeI* restriction enzymes. mPmlX1-tRFP, mPmlX2-tRFP, mPmlX3-tRFP, mPmlX4-tRFP, mPmlX6-tRFP and mPmlX~~K~~7-tRFP were constructed based on their exon composition from mPml1–3 and mPmlX~~K~~7 sequences along with two gene fragments (Tail1 and Tail2 (GeneScript)) using overlap extension PCR and *BamHI* and *NotI* restriction enzymes. mPmlX5-tRFP was constructed using *ApaI* and *SacI* restriction enzymes. mPml1 was introduced with a terminal stop codon and cloned into the pCMV6-AC-RFP vector by mutagenic PCR and blunt-end ligation. The mPml2, mPml3, mPmlX1, mPmlX2, mPmlX3, mPmlX4, mPmlX5, mPmlX6 and mPmlX~~K~~7-tRFP constructs were introduced with a terminal stop codon using KLD Enzyme Mix (NEB). Details on used plasmids, gene fragments and oligonucleotide primers are listed in the table below. All oligonucleotide primers were purchased from Integrated DNA Technologies and are summarized in **Supplementary Table S4**.

**Table S3 alt text:** Table summarizing the workflow for generation of murine PML isoform expression plasmids. Construction methods, commercial kits, gene fragments, templates, restriction enzymes and primers with their melting temperatures are included. Additional information on gene fragment sequences (5’–3’), template amounts and ratios, and accession numbers of commercial constructs is provided.

| **Plasmid** | **Construction** | **Primers (5’-3’)** | **Template** | **Enzymes** | **Additional info** |
| --- | --- | --- | --- | --- | --- |
| **pENTR/D-TOPO-mPml1** | pENTR™ Directional TOPO® Cloning | **RT-PCR**  mPml-ex1-F, mPml-ex9-R  T_m_ = 67 °C | MEF cells RNA | - | mPml-ex9-R reverse transcription |
| **pENTR/D-TOPO-mPmlX~~K~~7** | pENTR™ Directional TOPO® Cloning | **RT-PCR**  mPml-ex1-F, mPml-ex9-R  T_m_ = 67 °C | MEF cells RNA | - | mPml-ex9-R reverse transcription |
| **mPml1-tRFP** | NEBuilder® HiFi DNA Assembly Cloning Kit | **PCR1**  tRFP-vector-F, tRFP-vector-R  T_m_ = 69 °C | mPml3-tRFP | - | - |
|  |  | **PCR2**  mPml1-insert-F, mPml1-insert-R  T_m_ = 65 °C | pENTR/D-TOPO-mPml1 | - | - |
| **mPml2-tRFP** | DNA digestion | - | MG226141  mPml3-tRFP | *NotI*  *NdeI* | MG226141: pCMV6-AC-GFP vector (PS100010) encoding mPml2 (OriGene) |
| **mPml3-tRFP** | pCMV6-AC-RFP vector (OriGene; PS100034) encoding mPml3 | - | - | - | OriGene |
| **mPmlX1-tRFP** | Gene fragments PCR | **PCR1**  mPml-AllIs-F, mPmlX1-in-R  T_m_ = 70 °C | mPml2-tGFP | - | Tail1 **(5’-3’)**  CAGACACCAGAGGCACCCCAGACCTAGACCTCCCTTCTAAAAGCCAGAACCAGGAAGGCTTGCATAAAGAAGGTGTGGCCTCTGAGCTCTCAAGGGCCCCAGTTGCCACCAACCACCACCACAGGTGGCCTTATGGCTCTTCCTGGATGTTCGATTCATGGTGTTTTCCAGATAGCCCAGAGTTCCAGCTTAGCGGACCGACGCGTAC |
|  |  | **PCR2**  Tail1-in-F, Tail1-R  T_m_ = 70 °C | Tail1 |  |  |
|  | Full gene PCR | mPml-AllIs-F, Tail1-R  T_m_ = 70 °C | PCR1:PCR2 | - | 1:1 molar ratio |
|  | DNA digestion | - | Vector: mPml1-tRFP  Insert: mPmlX1 | *BamHI*  *NotI* | - |
| **mPmlX2-tRFP** | Gene fragments PCR | **PCR1**  mPml-AllIs-F, mPmlX1-in-R  T_m_ = 70 °C | mPml1-tRFP | - | - |
|  |  | **PCR2**  Tail1-in-F, Tail1-R  T_m_ = 70 °C | Tail1 |  |  |
|  | Full gene PCR | mPml-AllIs-F, Tail1-R  T_m_ = 70 °C | PCR1:PCR2 | - | 1:1 molar ratio |
|  | DNA digestion | - | Vector: mPml1-tRFP  Insert: mPmlX2 | *BamHI*  *NotI* | - |
| **mPmlX3-tRFP** | Gene fragments PCR | **PCR1**  mPml-AllIs-F, mPmlX3-in-R  T_m_ = 70 | mPml3-tRFP | - | Tail2 **(5’-3’)**  GGACACTTCTCTGAGCTTCATCCTGACCCTGACCCTAGCCTGTTGACTATACCTTCTGCTCCTGGTAAGAAACTGAAGCCCTTTTGGCGGTGTGGGAATTGTCAGCATCTGCTACCTAATTACAGATTTGTCTGCTGTTGGCACTTCCCTTCATCTGCCACTTCTGCCTGGTTGGAGGGGAAGTCTGTCTTCCTCAGCGGACCGACGCGTAC |
|  |  | **PCR2**  Tail2-in-F, Tail2-R  T_m_ = 70 °C | Tail2 |  |  |
|  | Full gene PCR | mPml-AllIs-F, Tail2-R  T_m_ = 70 °C | PCR1:PCR2 | - | 1:1 molar ratio |
|  | DNA digestion | - | Vector: mPml1-tRFP  Insert: mPmlX3 | *BamHI*  *NotI* | - |
| **mPmlX4-tRFP** | Gene fragments PCR | **PCR1**  mPml-AllIs-F, mPmlX4-in-R  T_m_ = 70 °C | mPml3-tRFP | - | - |
|  |  | **PCR2**  mPmlX4-in-F, mPmlX4-out-R  T_m_ = 70 °C | mPml3-tRFP |  |  |
|  | Full gene PCR | mPml-AllIs-F, mPmlX4-out-R  T_m_ = 70 °C | PCR1:PCR2 | - | 70 ng:30 ng |
|  | DNA digestion | - | Vector: mPml1-tRFP  Insert: mPmlX4 | *BamHI*  *NotI* | - |
| **mPmlX5-tRFP** | DNA digestion | - | mPml1-tRFP  mPml3-tRFP | *SacI*  *ApaI* | - |
| **mPmlX6-tRFP** | Gene fragments PCR | **PCR1**  mPml-AllIs-F, mPmlX4-in-R  T_m_ = 70 °C | mPml1-tRFP | - | - |
|  |  | **PCR2**  mPmlX4-in-F, mPmlX4-out-R  T_m_ = 70 °C | mPml1-tRFP |  |  |
|  | Full gene PCR | mPml-AllIs-F, mPmlX4-out-R  T_m_ = 70 °C | PCR1:PCR2 | - | 70 ng:30 ng |
|  | DNA digestion | - | Vector: mPml1-tRFP  Insert: mPmlX6 | *BamHI*  *NotI* | - |
| **mPmlX~~K~~7-tRFP** | Gene fragments PCR | **PCR1**  mPml-AllIs-F, mPmlX~~K~~7-in-R  T_m_ = 66 °C | mPml1-tRFP | - | - |
|  |  | **PCR2**  mPmlX~~K~~7-in-F, mPml-AllIs-R  T_m_ = 72 °C | mPml1-tRFP |  |  |
|  | Full gene PCR | mPml-AllIs-F, mPml-AllIs-R  T_m_ = 70 °C | PCR1:PCR2 | - | 35 ng:65 ng |
|  | DNA digestion | - | Vector: mPml1-tRFP  Insert: mPmlX~~K~~7 | *BamHI*  *NotI* | - |
| **mPml1-S** | Site Directed PCR Mutagenesis | tRFP-vector-F, mPml-ex9-R  T_m_ = 67 °C | mPml1-tRFP |  |  |
| **mPml2-S** | Site Directed PCR Mutagenesis | mPml-AllIs-KLD-F, mPmlX~~K~~7-KLD-R  T_m_ = 71 °C | mPml2-tGFP | - | - |
| **mPml3-S** | Site Directed PCR Mutagenesis | mPml-AllIs-KLD-F, mPml3-KLD-R  T_m_ = 68 °C | mPml3-tRFP | - | - |
| **mPmlX1-S** | Site Directed PCR Mutagenesis | mPml-AllIs-KLD-F, mPmlX2-KLD-R  T_m_ = 68 °C | mPmlX1-tRFP | - | - |
| **mPmlX2-S** | Site Directed PCR Mutagenesis | mPml-AllIs-KLD-F, mPmlX2-KLD-R  T_m_ = 68 °C | mPmlX2-tRFP | - | - |
| **mPmlX3-S** | Site Directed PCR Mutagenesis | mPml-AllIs-KLD-F, mPmlX3-KLD-R  T_m_ = 68 °C | mPmlX3-tRFP | - | - |
| **mPmlX4-S** | Site Directed PCR Mutagenesis | mPmlX4-KLD-F, mPmlX4-KLD-R  T_m_ = 72 °C | mPmlX4-tRFP | - | - |
| **mPmlX5-S** | Site Directed PCR Mutagenesis | mPml-AllIs-KLD-F, mPml3-KLD-R  T_m_ = 68 °C | mPmlX5-tRFP | - | - |
| **mPmlX6-S** | Site Directed PCR Mutagenesis | mPml-AllIs-KLD-F, mPmlX4-KLD-R  T_m_ = 72 °C | mPmlX6-tRFP | - | - |
| **mPmlX~~K~~7-S** | Site Directed PCR Mutagenesis | mPml-AllIs-KLD-F, mPmlX~~K~~7-KLD-R  T_m_ = 71 °C | mPmlX~~K~~7-tRFP | - | - |

**Table S4:** Oligonucleotide primers used for plasmids construction.

**Table S4 alt text:** Table listing primers used for construction of murine PML isoform expression plasmids, including their sequences (5’–3’).

| **Primer** | **Sequence (5’-3’)** |
| --- | --- |
| mPml-F | CACCATGGAAACTGAACCAGTTTCCGT |
| mPml-R | GGCCAGGCATCCCTTACTTT |
| tRFP-vector-F | AGCGGACCGACGCGTACG |
| tRFP-vector-R | CTGCTCAAGGTCCTCAGGCG |
| mPml1-insert-F | ACGCCTGAGGACCTTGAG |
| mPml1-insert-R | CGCGTACGCGTCGGTCCGCTGGCCAGGCATCCCTTAC |
| mPml-AllIs-F | CTAAGGATCCGGTACCGAGGAGATC |
| mPml-AllIs-R | CATAGCGGCCGCGTACGCGTCGGTCCGCTG |
| mPmlX1-in-R | CTCTGGTGTCTGTTTCAGCTTTGCCTTCTGCCC |
| mPml3-in-R | CAGAGAAGTGTCCCAGATTCTCGGTGTCCGAATCCTC |
| Tail1-in-F | GGCAAAGCTGAAACAGACACCAGAGGCACCC |
| Tail1-R | CATGCGGCCGCGTACGCGTCGGTCCGCTAAG |
| Tail2-in-F | CGAGAATCTGGGACACTTCTCTGAGCTTCATCCTG |
| Tail2-R | CATGCGGCCGCGTACGCGTCGGTCCGCTGAG |
| mPmlX4-in-F | CCCAGAAAATTAGCCAGCTGGCCGCGGAGCGGACCGACGCGTAC |
| mPmlX4-in-R | GCCAGCTGGCTAATTTTCTGGGCAGATTCTCGGTGTCCGAATCCT |
| mPmlX4-out-R | TCATCTGTGCCCCAGTTTGCTAG |
| mPmlX~~K~~7-in-F | TCCATTGCGATATTGATGCAGCTGTAGCCAGCCC |
| mPmlX~~K~~7-in-R | CTACAGCTGCATCAATATCGCAATGGAGATGGCTGTG |
| mPml-AllIs-KLD-F | TAGAGCGGACCGACGCGTAC |
| mPmlX4-KLD-F | TGAAGCGGACCGACGCGTAC |
| mPmlX~~K~~7-KLD-R | GGCCAGGCATCCCTTACTTTCAG |
| mPml3-KLD-R | ATTTTTTTCATTGTCAATCTTGAGGTCAAAGAAAACC |
| mPmlX4-KLD-R | CCGCGGCCAGCTGGCTAATTTTC |
| mPmlX2-KLD-R | AAGCTGGAACTCTGGGCTATCTGG |
| mPmlX3-KLD-R | GAGGAAGACAGACTTCCCCTCCAAC |
| mPml-ex1-F | CACCATGGAAACTGAACCAGTTTCCGT |
| mPml-ex9-R | CTAGGCCAGGCATCCCTTACTTT |

**
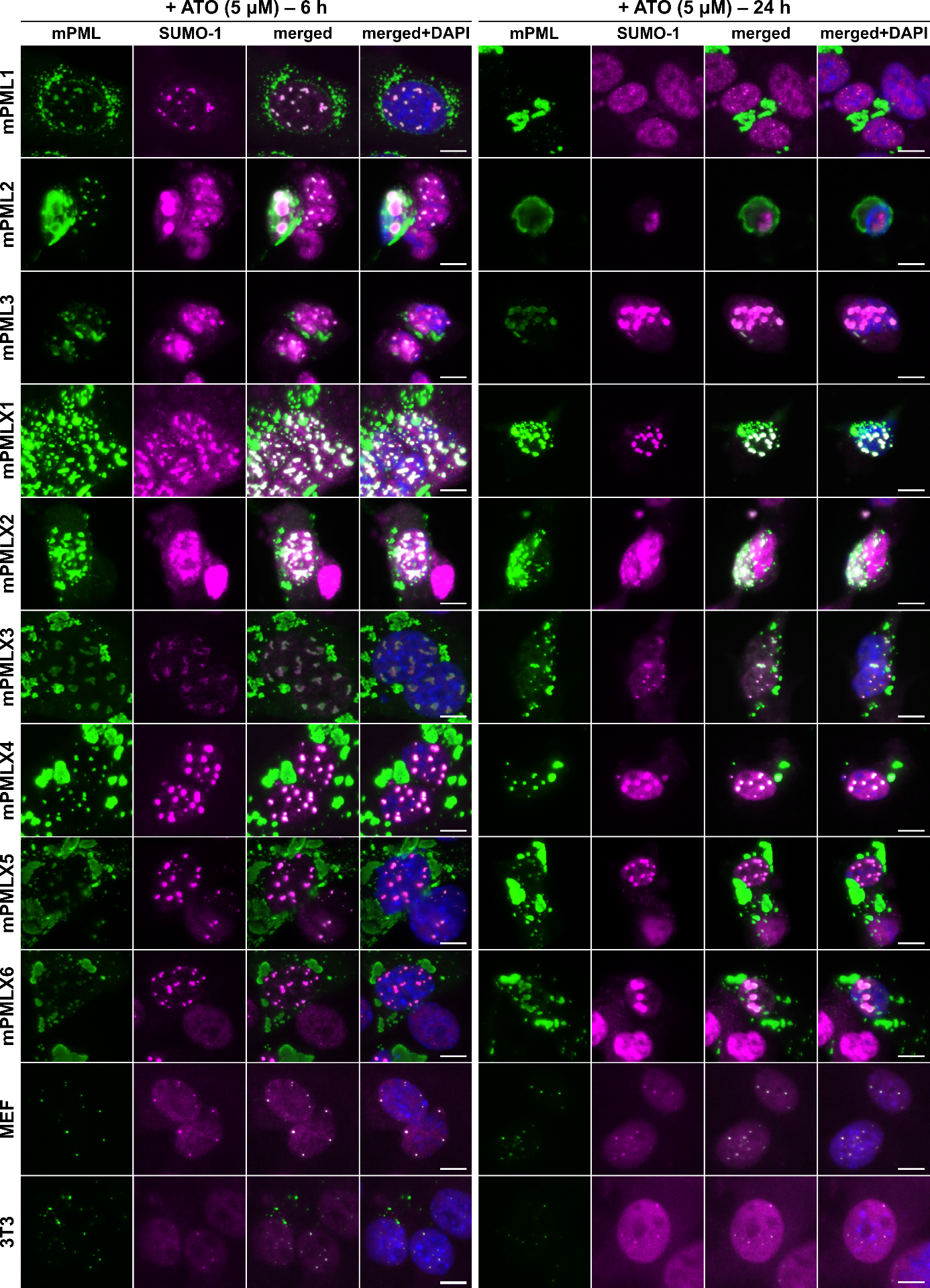
**

**Figure S6.** The effect of arsenic treatment on mPML NB dynamics. *Pml^−/−^* MEFs transiently expressing individual mPML isoforms or control *Pml^+/+^* MEFs or 3T3 cells were treated with 5 µM ATO for 6 and 24 h. Subsequently, the cells were fixed and stained using specific antibodies against mPML (green) and SUMO-1 (magenta) proteins. Cell nuclei were visualized by DAPI staining (blue). Representative fluorescence microscopy images are shown. Scale bar, 10 μm.

**Figure S6 alt text:** Fluorescent microscopy images, including single-channel (n=3) and merged views of murine PML and SUMO-1 proteins, showing the effect of arsenic trioxide treatment on PML nuclear bodies formed by individual murine PML isoforms at different times post-treatment.

**
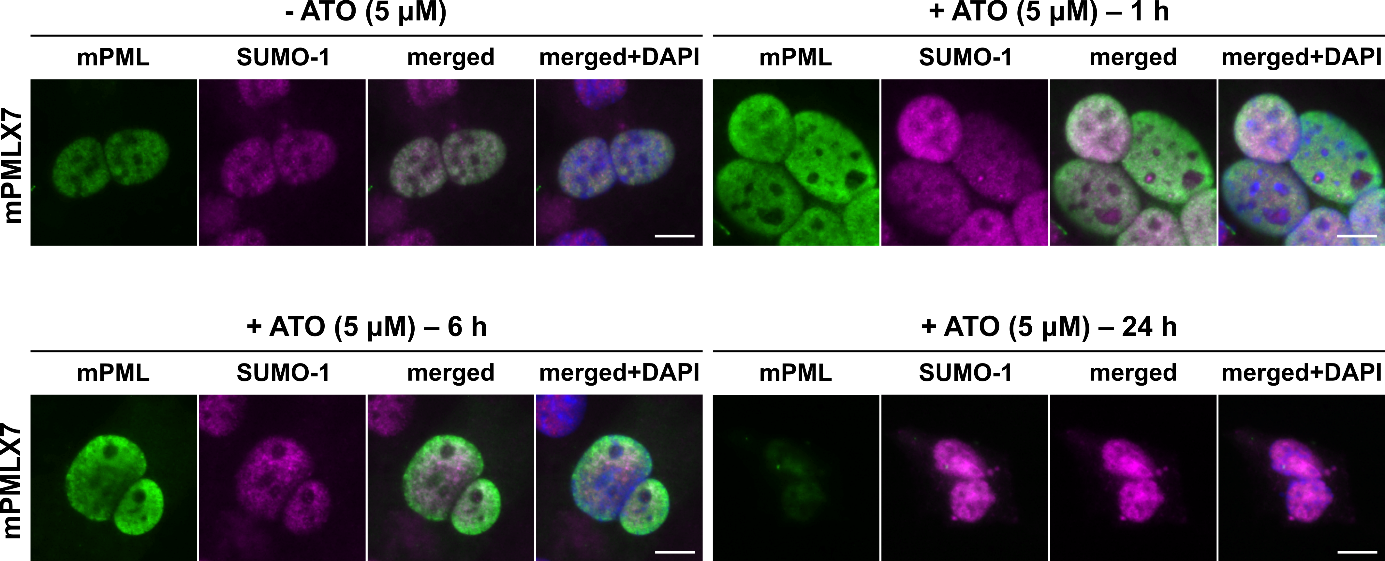
**

**Figure S7.** The effect of arsenic treatment on mPML NB dynamics. *Pml^−/−^* MEFs transiently expressing mPMLX~~K~~7 isoform were mock-treated or treated with 5 µM ATO for 1, 6, and 24 h. Subsequently, the cells were fixed and stained using specific antibodies against mPML (green) and SUMO-1 (magenta) proteins. Cell nuclei were visualized by DAPI staining (blue). Representative fluorescence microscopy images are shown. Scale bar, 10 μm.

**Figure S7 alt text:** Fluorescent microscopy images, including single-channel (n=3) and merged views of murine PML and SUMO-1 proteins, visualizing the effect of arsenic trioxide treatment on PML nuclear bodies formed by the PMLX7 isoform.

**
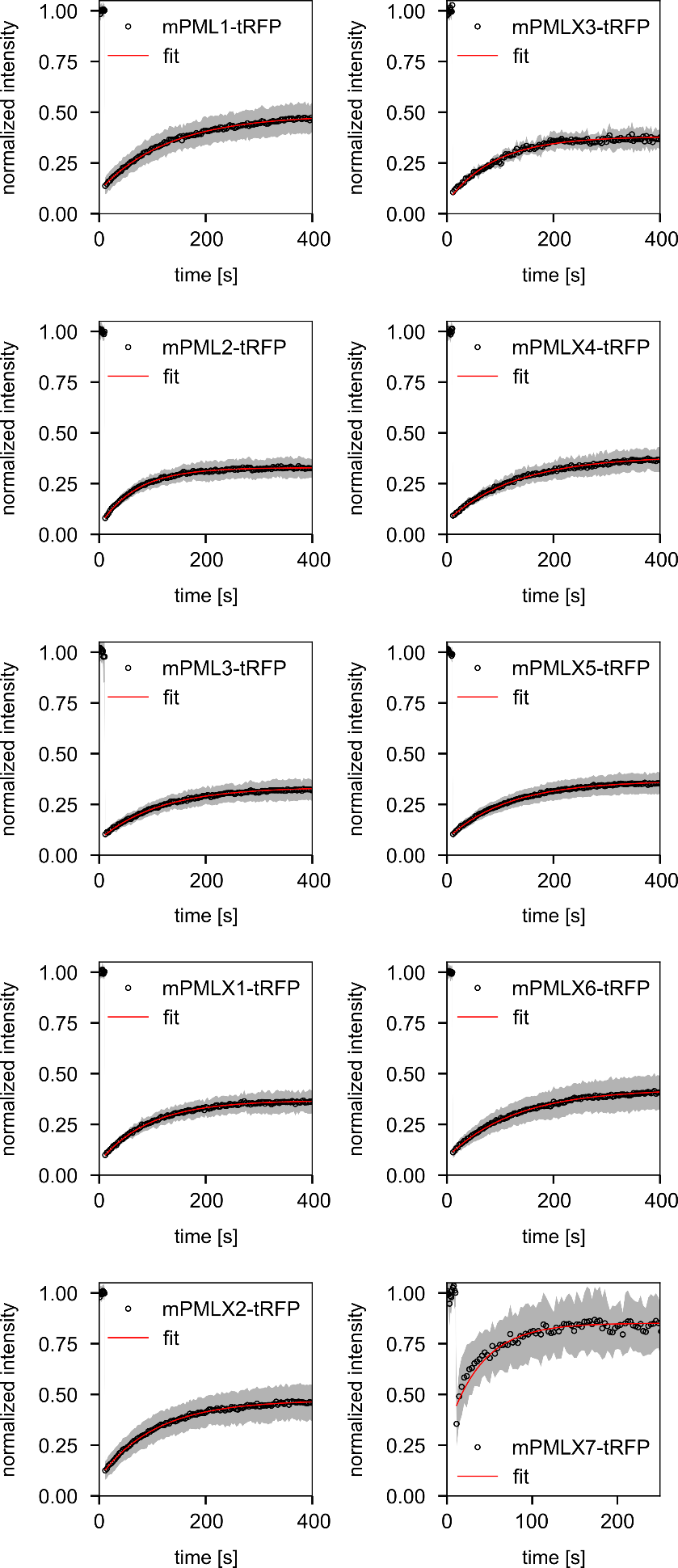
**

**Figure S8**. Exchange of individual mPML isoforms at endogenous mPML NBs. Isoform-averaged fluorescence recovery traces. Black circles indicate the mean across all recorded traces for the isoform, with the gray shaded area denoting ± one standard deviation. The red curve represents a single-exponential fit using the averaged parameters from individual fits.

**Figure S8 alt text:** A multi-panel figure containing ten graphs showing the dynamic turnover of individual murine PML isoforms at nuclear bodies using fluorescence recovery after photobleaching (FRAP). In each graph, the x-axis represents time in seconds and the y-axis represents normalized fluorescence intensity

**
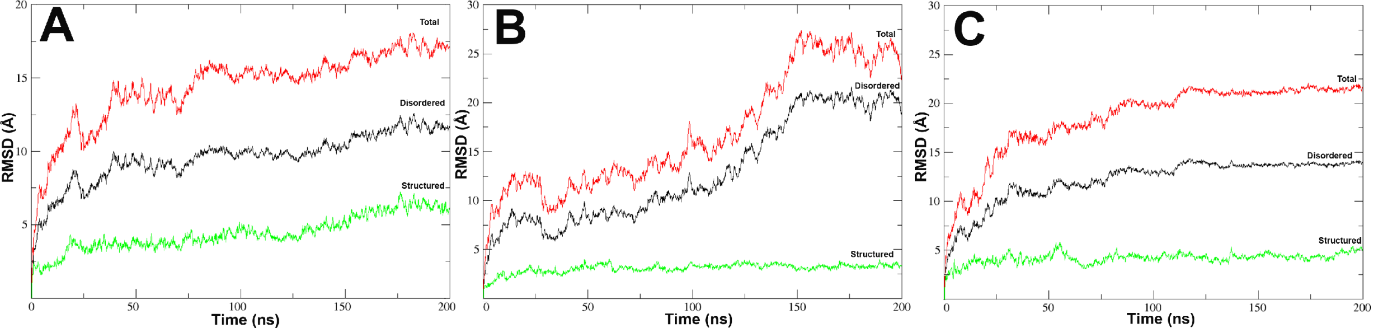
**

**Figure S9:** Best-fit mass-weighted C_a_ RMSD plots of (**A**) Zn-free, (**B**) Zn_(1)_ and (**C**) Zn_(2)_ structures along the 200-ns of the MD production runs. The plots are split into structured (lime), disordered (black) and total (red) parts and use the first frame of the run as reference.

**Figure S9 alt text:** A multi-panel figure containing three Root-Mean-Square Deviation (RMSD) plots derived from molecular dynamics simulations of three mPML isoform 7 variants: zinc-bound at position 1, zinc-bound at position 2, and a zinc-free control. In each graph, the x-axis represents simulation time and the y-axis represents the RMSD value.

**
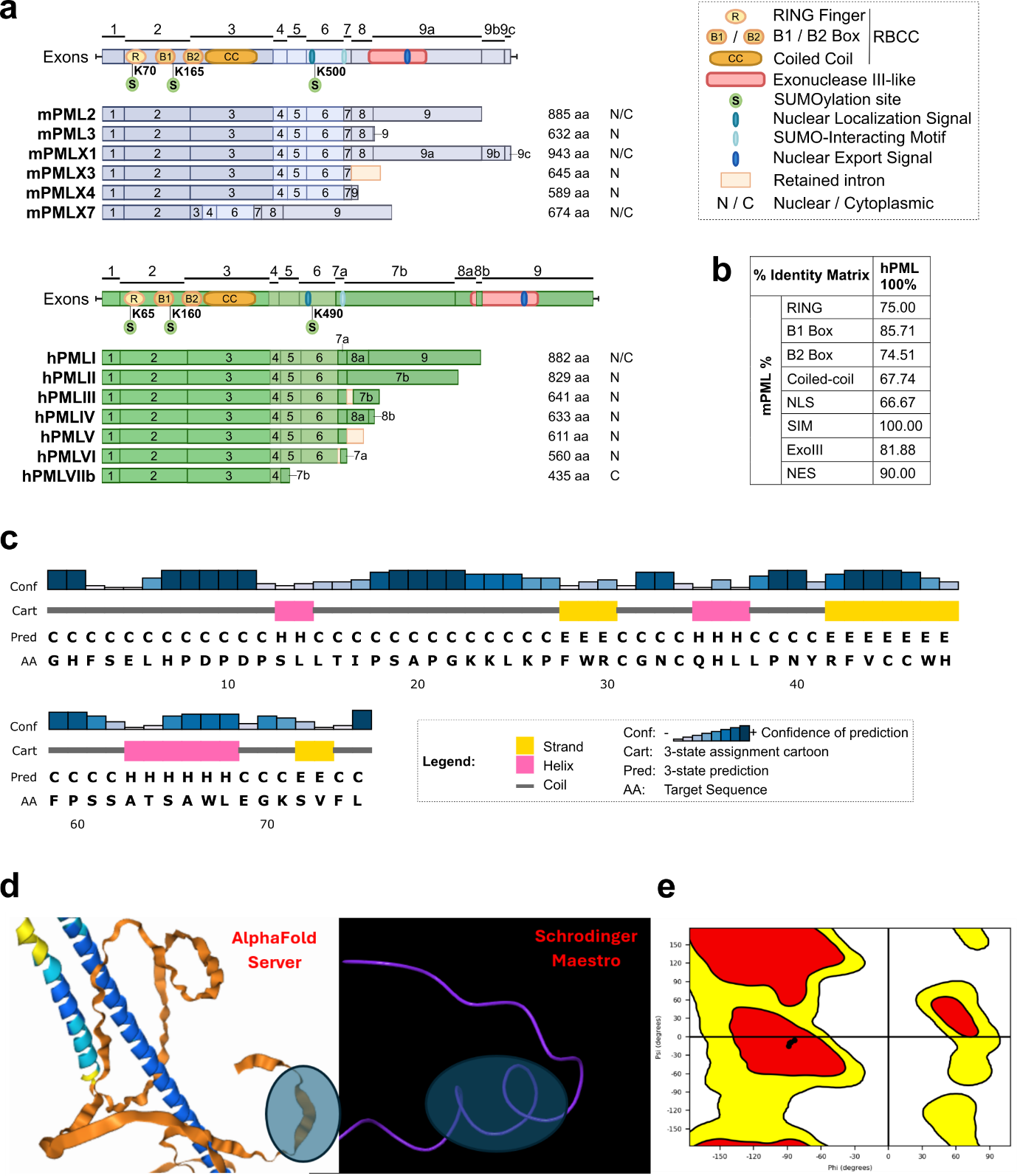
**

**Figure S10:** Comparison of the *PML* gene structure and PML protein isoforms across different mouse and human homologs. **(a)** Schematic representation of exon organization, domain composition, isoform length and possible cellular localization. Only translated regions (from the start to the stop codon) and isoforms containing putative exon 5 for “a–c” subgroups as suggested in[33] of each isoform are shown. **(b)** Analysis of sequence similarities of domains and motifs among mouse and human PML proteins using EMBL-EBI Clustal Omega Percent Identity scores, measuring the number of identical residues relative to the alignment length. **(c)** Predicted structural α-helix within the mPMLX3 intronic sequence. The sequence (^633^ATSAWL^638^) is shown to form near-helical structure as hinted by the PSIPRED 4.0 Protein Analysis Workbench with medium-high confidence and **(d)** AlphaFold and pyMol/Maestro viewers. **(e)** The Ramachandran plot shows that all six residues of the sequence lie within the right-handed alpha-helix region.

**~~Figure S10:~~** ~~Predicted structural α-helix within the mPMLX3 intronic sequence. The sequence (~~^~~633~~^~~ATSAWL~~^~~638~~^~~) is shown to form near-helical structure as hinted by the (a) PSIPRED 4.0 Protein Analysis Workbench with medium-high confidence and (b) AlphaFold and pyMol/Maestro viewers. (c) The Ramachandran plot shows that all six residues of the sequence lie within the right-handed alpha-helix region.~~

**Figure S10 alt text:** A multi-panel figure comparing mouse and human PML protein isoforms. Panel (a) provides a schematic of exon composition and protein domain architecture (b) displays a similarity matrix table showing the percentage of domain conservation between species. Panels (c–e) utilize structural prediction tools to analyse features within mPMLX3: Panel (c) shows a PSIPRED output, where alpha-helices, beta-strands, and coils are mapped beneath the amino acid sequence. Panel (d) presents 3D structural models generated to highlight the spatial arrangement of a predicted alpha-helix. Panel (e) displays a Ramachandran plot, plotting Phi vs. Psi angles.

**
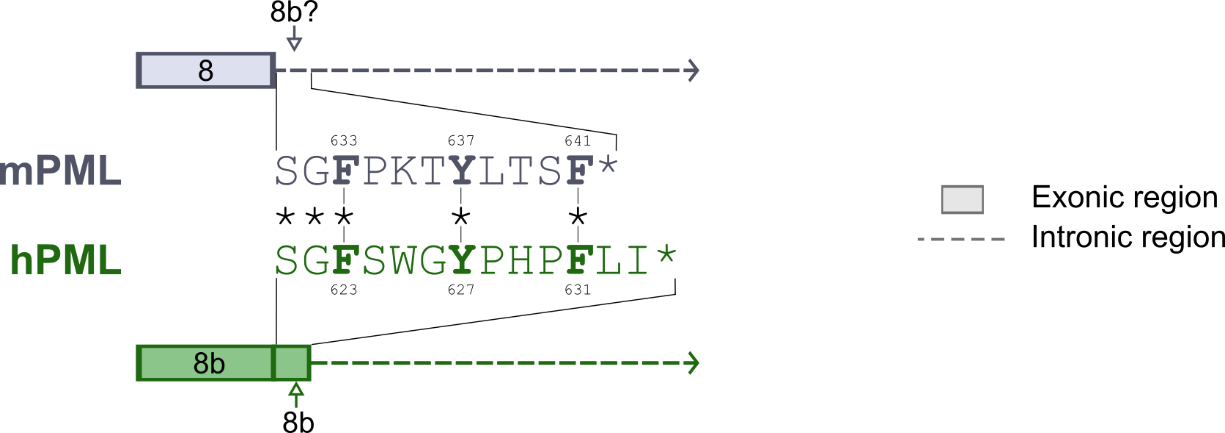
**

**Figure S11:** Visualization of amino-acid sequence conservation between human exon 8b and murine 8/9 intronic region.

**Figure S11 alt text:** A detailed schematic comparing specific regions of the mouse and human PML genes. The diagram illustrates the transition from the exonic region (solid boxes) to the intronic region (dashed lines with arrows). The figure displays the amino acids encoded by the human exon 8b region while highlighting the possibility that the corresponding intronic region may be retained in the mouse exon 8 variant. Bold fonts and asterisks highlight conserved residues between the two species.

**Supplementary methodology**

**Detection of endogenous mPML transcripts by Oxford Nanopore Technology (ONT) Sequencing**

**RNA isolation**

Total RNA was isolated from overnight culture of *Pml^+/+^* MEFs seeded in a 6-well plate using the NucleoSpin RNA Kit (Macherey-Nagel) according to the manufacturer’s protocol. The concentration was measured using NanoDrop 3000 and RNA purity was assessed by RIN analysis on the 2100 Bioanalyzer (Agilent). RNA quality was further evaluated using the Qubit RNA BR Assay Kit (Thermo Fisher Scientific) and the Bioanalyzer RNA 6000 Nano Kit (Agilent).

**Library preparation**

The sequencing library was prepared using the cDNA-PCR Sequencing V14 – Barcoding Kit (SQK-PCB114.24) (ONT) according to manufacturer’s instructions, where full-length RNA is reverse transcribed with cDNA RT adapter and subsequently sequenced using the appropriate flow cell and device. Library quality was assessed using E-Gel^™^ EX Agarose Gels, 1% (Invitrogen) and the Qubit 1X dsDNA High-Sensitivity Assay Kit (Thermo Fisher Scientific).

**Nanopore Sequencing**

Sequencing run was performed using the cDNA-PCR Sequencing V14 – Barcoding Kit (SQK-PCB114.24) on a PromethION Flow Cell with R10.4.1 chemistry (FLO-PRO114M) on the PromethION 2 device (ONT). The sequencing run generated 9,239,441 reads, with a median read length of 2513 bp and a GC content of 48 %.

**Data analysis**

Data analysis was performed in three phases. In phase I, raw sequencing reads were mapped to potential transcript sequences including previously confirmed isoforms (mPML1–mPML3), computationally predicted isoforms (mPMLX1–X6), and the potential novel isoform mPMLX7. Extracted reads were processed using the Psychopper programme (ONT) to trim of ONT adapters and baracodes and to orient reads in the 5’–3’ direction. Quality control of the individual reads was performed using the FASTQC program (Andrews, S. (2010). FastQC: A Quality Control Tool for High Throughput Sequence Data. Available online at: http://www.bioinformatics.babraham.ac.uk/projects/fastqc/.) and the reads were then realigned to transcript sequences from the NCBI database using the Geneious Prime software with Minimap2 aligner tool, and visualized using the Integrative Genomics Viewer (IGV).

In the second phase, the presence of individual transcripts was analyzed by aligning reads separately to each NCBI database-annotated transcript. Only reads starting no later than the exon1/2 border (nucleotide (nt) 193/194 for mPML2, GeneBank accession number NM 178087.5) and covering the full sequence till the polyadenylation signal were included in downstream analysis.

In phase III, only reads that have no insertion and/or deletion longer than 10 nt were filtered out. However, the sequence on the boundary of exons 8 and 9 had to align to reference sequence with insertion and/or deletion no longer than 2 nt, as the two alternative 3’ splice sites differ only by 5 nt.
